# Supplementary material for: Development of allosteric and selective CDK2 inhibitors for contraception with negative cooperativity to cyclin binding
Source: Nat Commun. 2023 Jun 3;14:3213. doi: 10.1038/s41467-023-38732-x (PMC10239507; doi:10.1038/s41467-023-38732-x)
Supplement: Supplementary file 1 — Supplementary Information [file 41467_2023_38732_MOESM1_ESM.pdf]

Development of allosteric and selective CDK2 inhibitors for contraception with negative cooperativity to cyclin binding

Supplementary Information

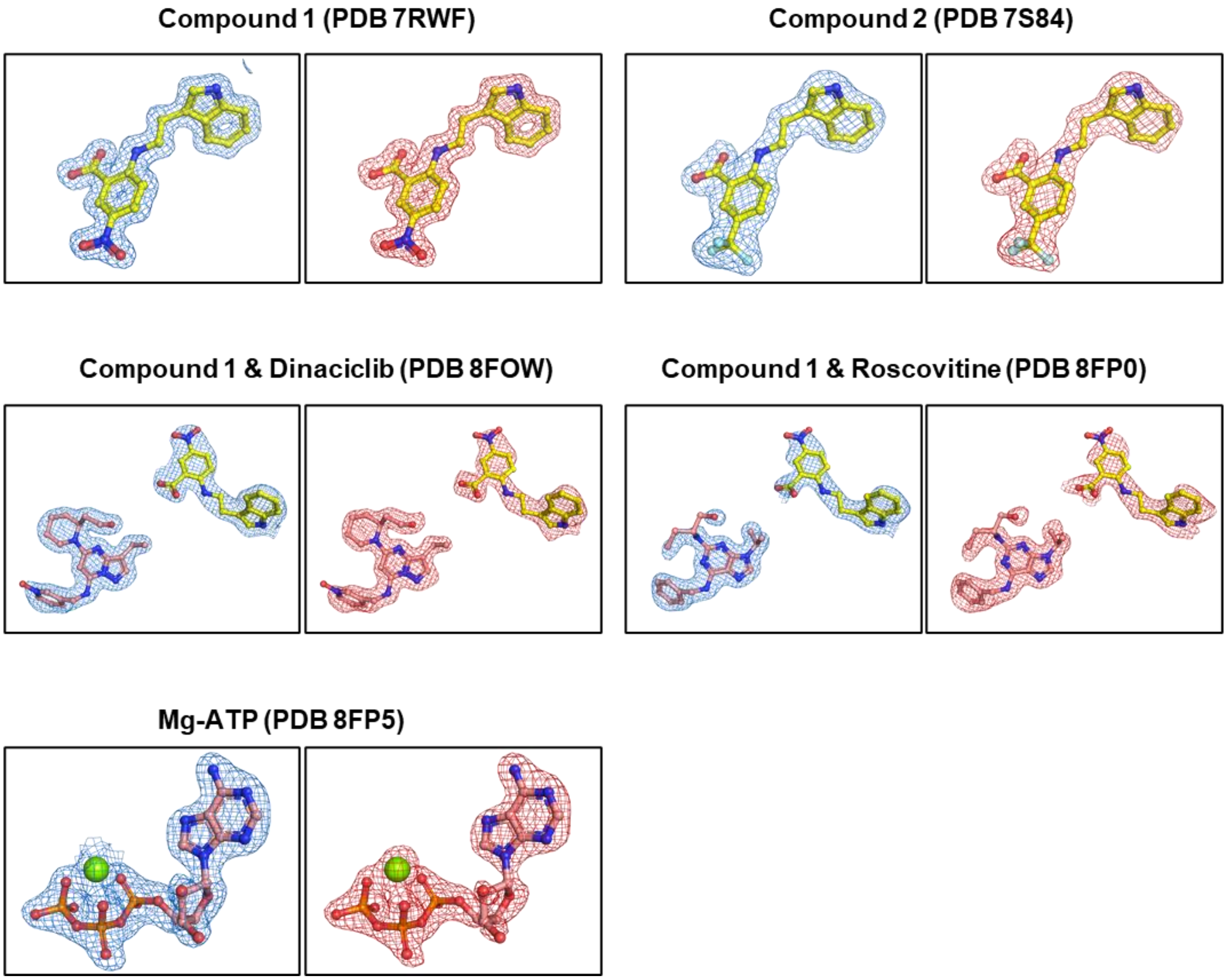

Supplementary Fig. 1. **Electron density maps of allosteric ligands of CDK2.** The 2Fo-Fc density map upon refinement with ligand is shown in blue (contoured at 1σ). The Fo-Fc density map upon refinement omitting the ligand is shown in red (contoured at 3σ).

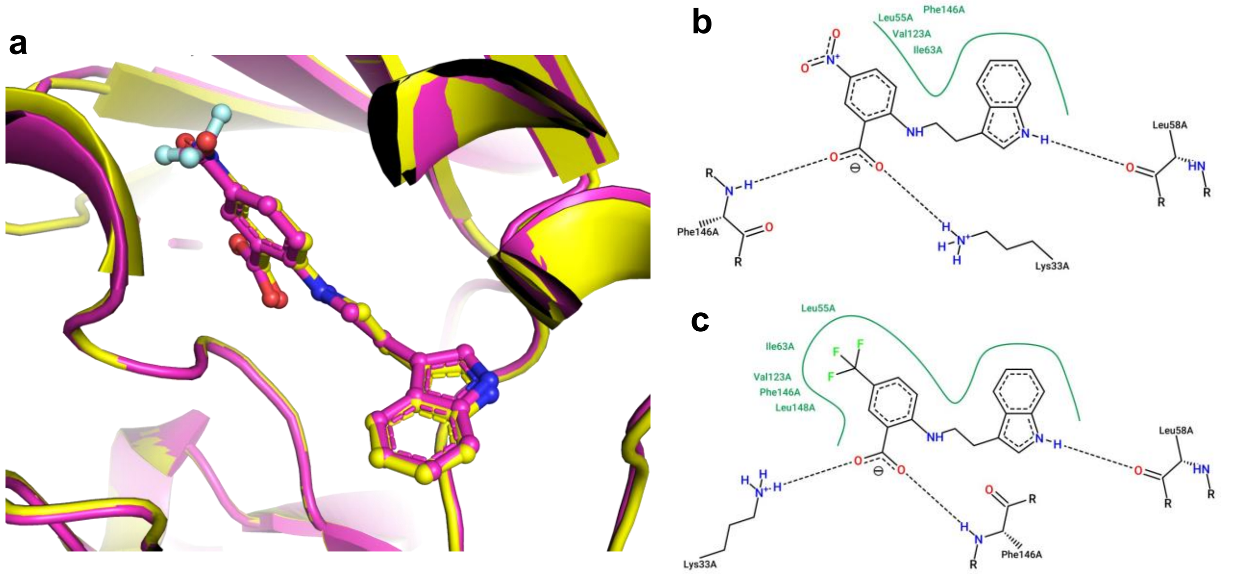

Supplementary Fig. 2. **Comparison of compounds 1 and 2 binding to CDK2. a** Superposition of allosteric ligands bound to CDK2. The structure of CDK2 with compound **1** is shown in yellow, the structure with compound **2** is shown in magenta. 2D interaction diagrams of CDK2 with compound **1** (**b**) and compound **2** (**c**) as computed by Poseview.[1]

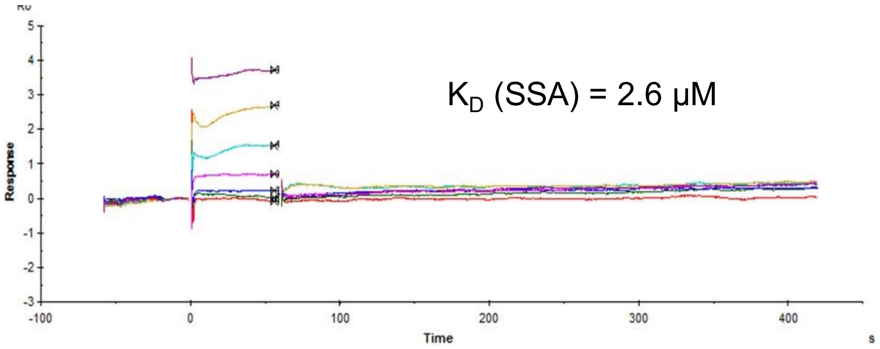

Supplementary Fig. 3. **SPR replicate measuring compound 1 binding to CDK2.** Compound **1** binds to CDK2 with a K_D = 2.6 µM in this replicate with fast association, fast dissociation kinetics as determined by SPR. The other replicate is shown in Fig. 1f in the manuscript.

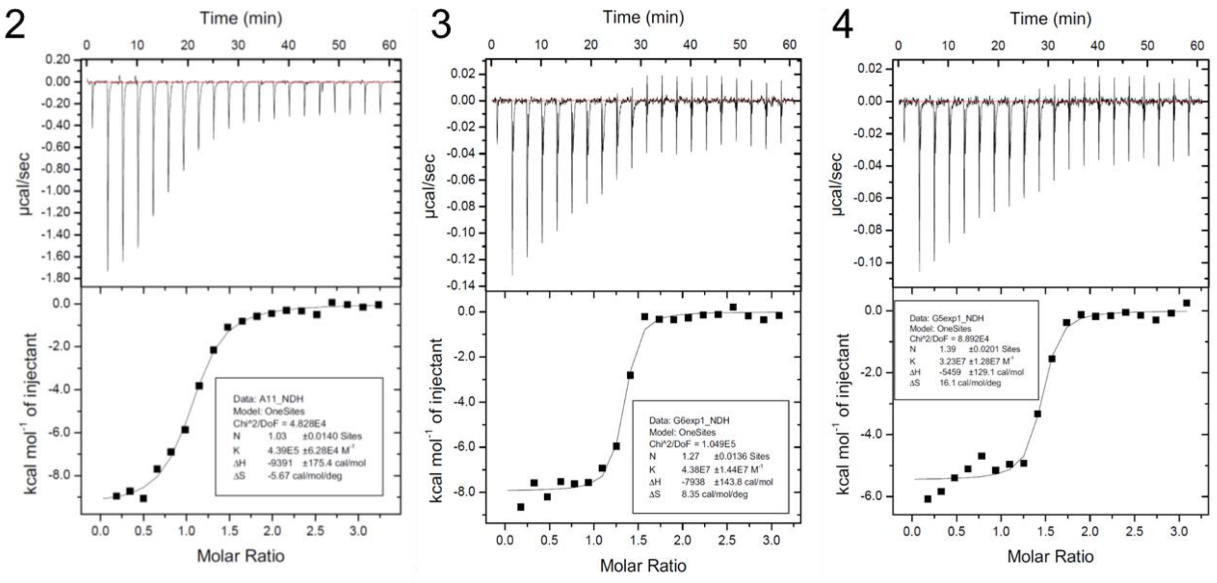

Supplementary Fig. 4. **ITC traces of 2, 3, and 4 against CDK2.** Results summarized in Table 1. Each ITC experiment was performed once.

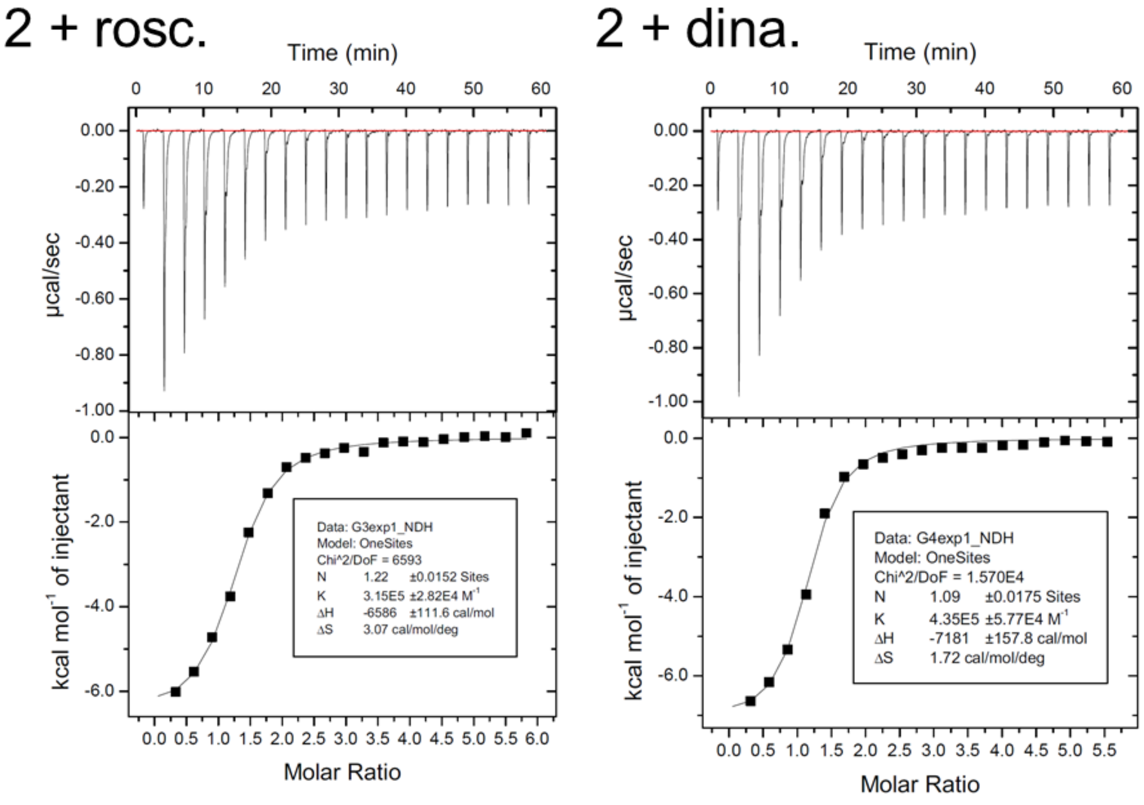

Supplementary Fig. 5. **ITC traces of 2 into CDK2 in the presence of roscovitine (rosc.) and dinaciclib (dina.).** Each ITC experiment was performed once.

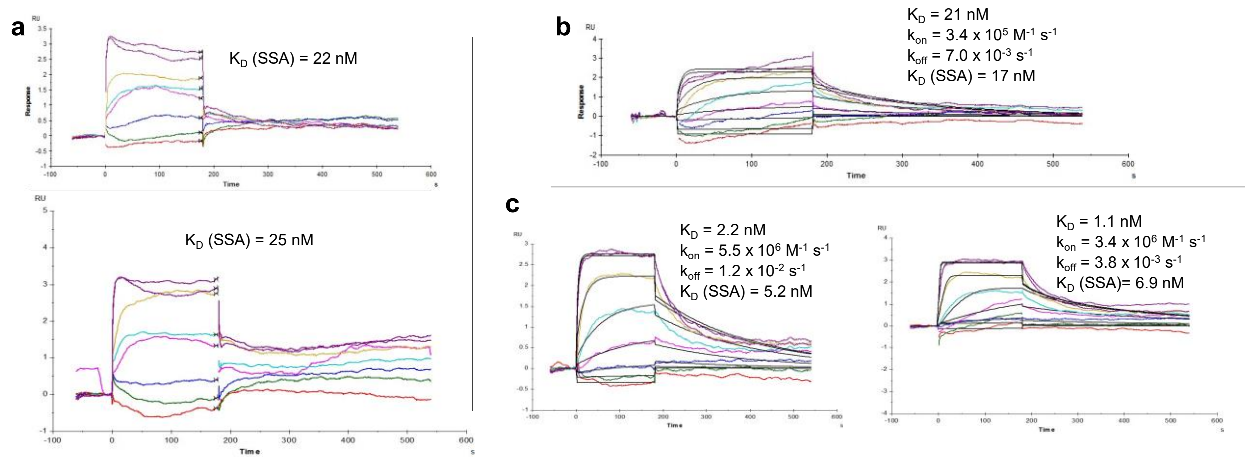

Supplementary Fig. 6. **Binding kinetics of compounds 3-5 towards CDK2. a** The SPR traces of **4** into CDK2. **b** The second replicate of **5** into CDK2. **c** The SPR traces of **3** into CDK2. The other replicate for **5** is shown in Fig. 2e in the manuscript. All SPR experiments done in 2 independent replicates for each compound.

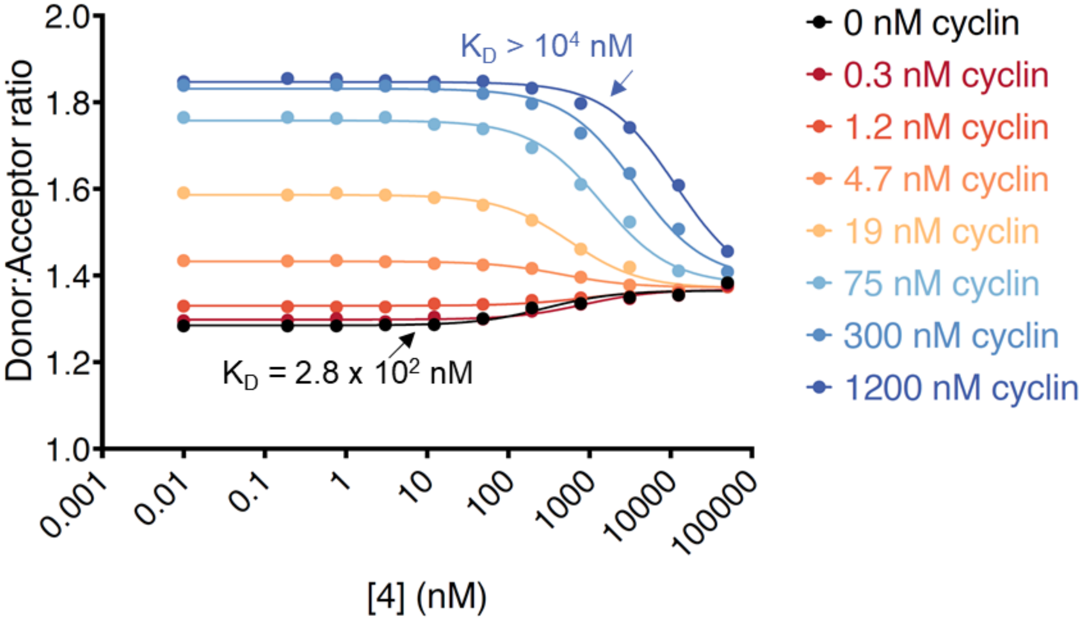

Supplementary Fig. 7. **Compound 4 demonstrates a negatively cooperative relationship with cyclin binding in CDK2.** Experiment performed once. Source data are provided as a Source Data file.

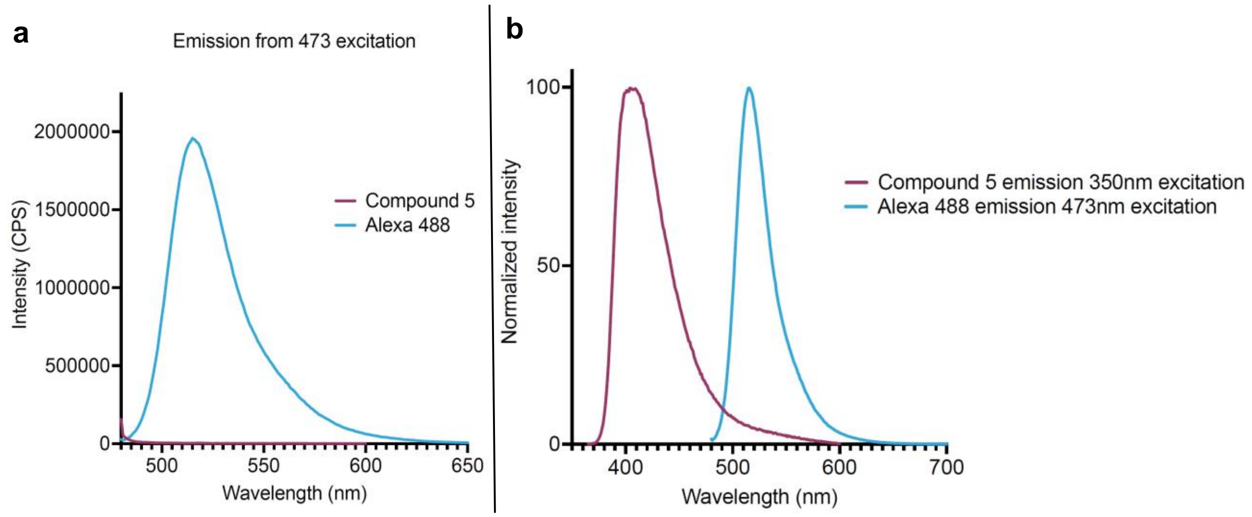

Supplementary Fig. 8. **Compound 5 does not show fluorescence interference with the Alexa 488 dye used in the FRET assay. a** Excited at the 473 nm wavelength used for the FRET experiments, compound **5** (10 µM, purple) shows no appreciable fluorescence in this emission range while Alexa 488 dye (5 nM, blue) has a predictable emission spectrum centered at ~520 nm. **b** Overlay of individually normalized fluorescent emission spectra of both compound **5** (purple) excited at 350 nm and Alexa 488 dye (blue) excited at 473 nm.

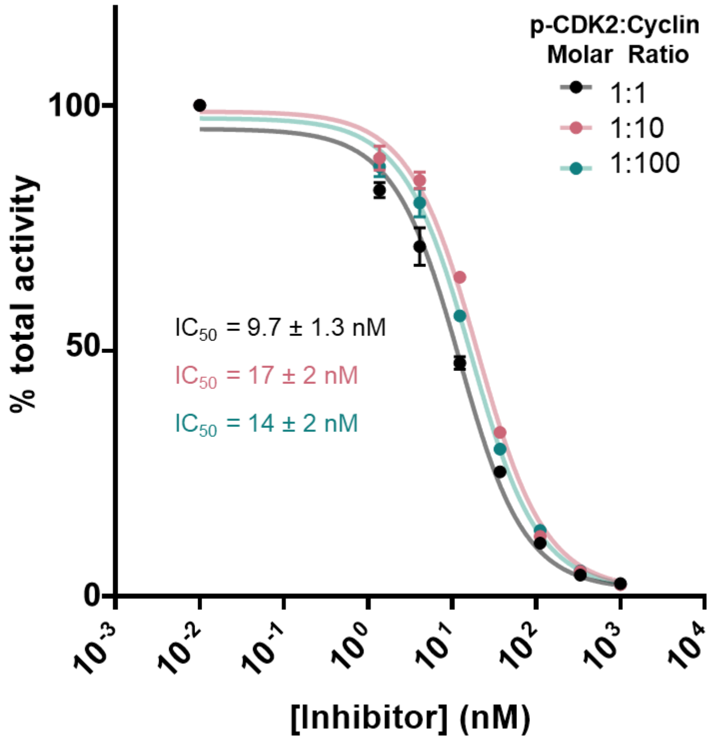

Supplementary Fig. 9. **Dinaciclib inhibits CDK2 largely independent of cyclin concentration.** Data points represent the mean value and error bars represent SEM for n=3 independent replicates for each condition. Source data are provided as a Source Data file.

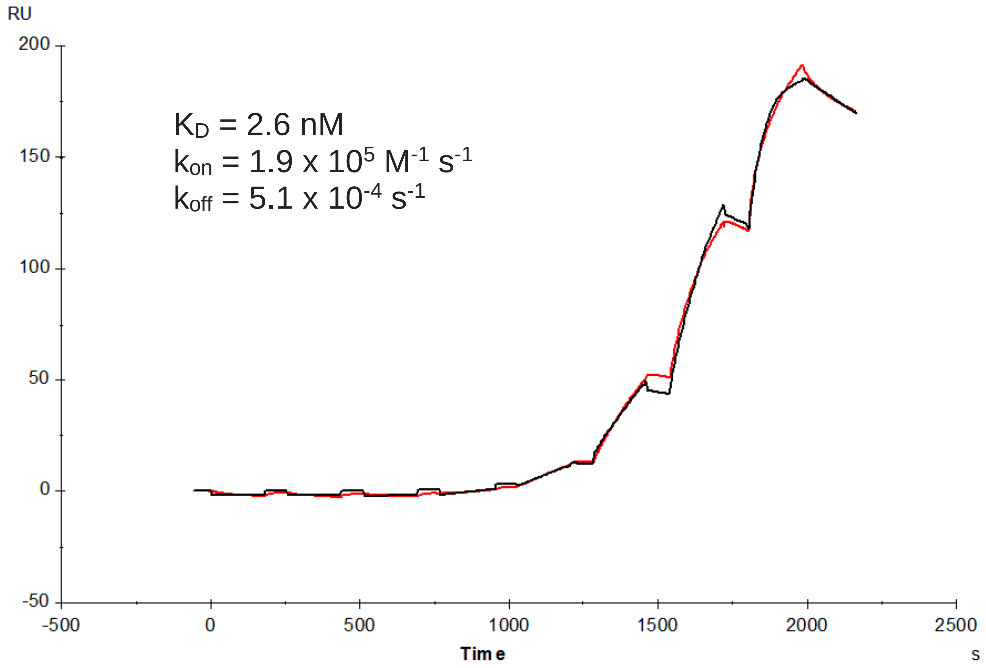

Supplementary Fig. 10. **SPR replicate measuring cyclin binding to CDK2.** The other replicate is shown in Fig. 2f in the manuscript. Experiment was performed twice in independent replicates.

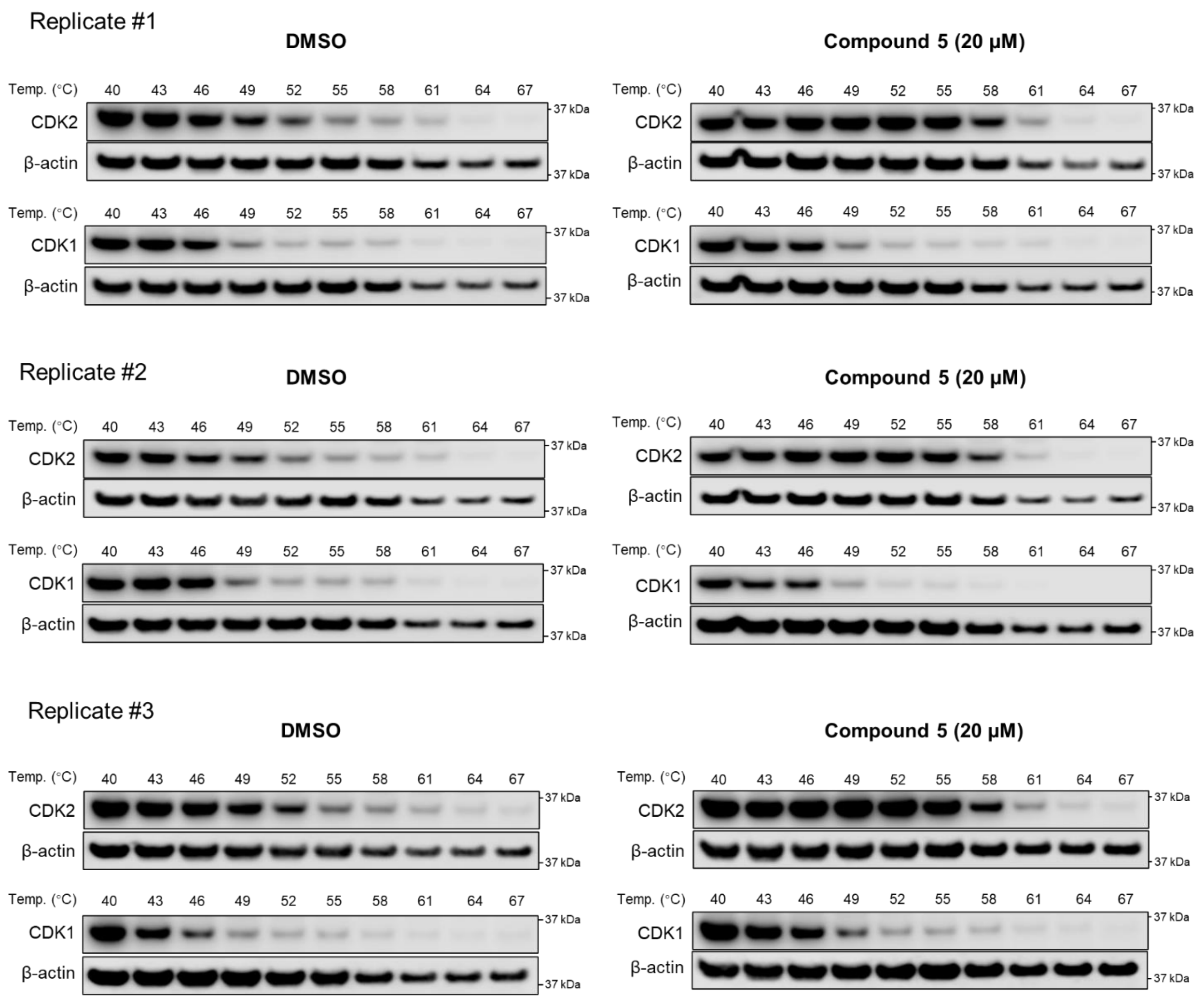

Supplementary Fig. 11. **Triplicate CETSA data for DMSO (control) and compound 5 treated Jurkat cells.** Numbers shown above bands on blots indicate the temperature of the experiment (°C). Source data are provided as a Source Data file.

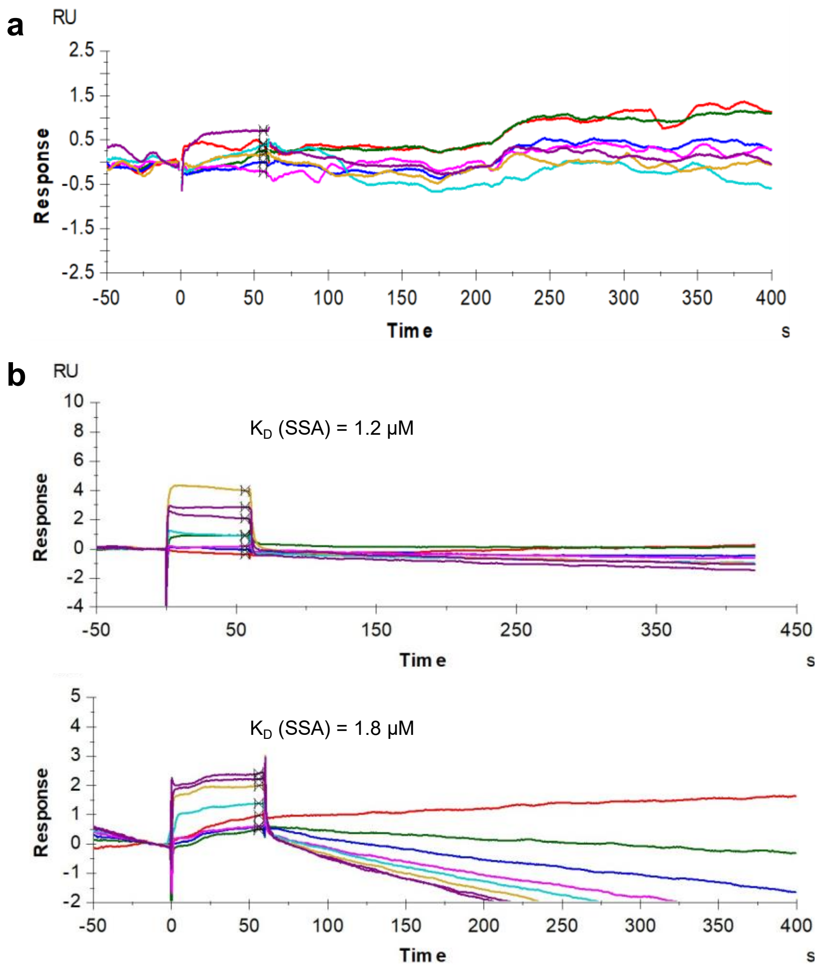

Supplementary Fig. 12. **Small molecule binding to CDK1. a** In an SPR assay, compound **5** does not bind CDK1. **b** In contrast, dinaciclib binds CDK1 in the SPR assay with an affinity similar to its published value (published value[2]: K_D (SSA) = 1.8 ± 0.2 µM). Experiment was performed twice in independent replicates.

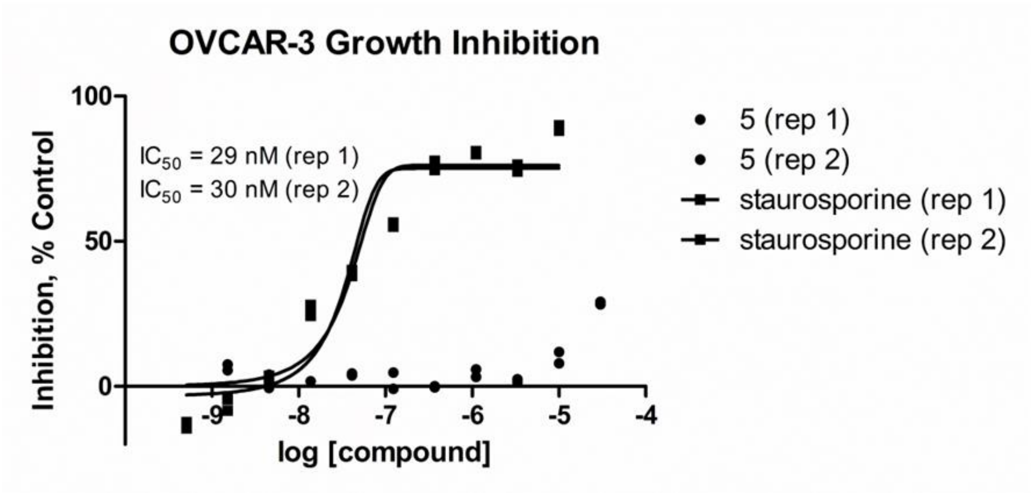

Supplementary Fig. 13. **Cytotoxicity of compound 5.** Compound **5** shows minimal cytotoxicity against a cell line with cyclin E1 overexpression and dependent on CDK2 activity (OVCAR-3), in contrast to the toxic kinase inhibitor staurosporine with IC_{50} values in the low nanomolar range. Two technical replicates (rep) for each compound were performed. Data provided by Pharmaron. Source data are provided as a Source Data file.

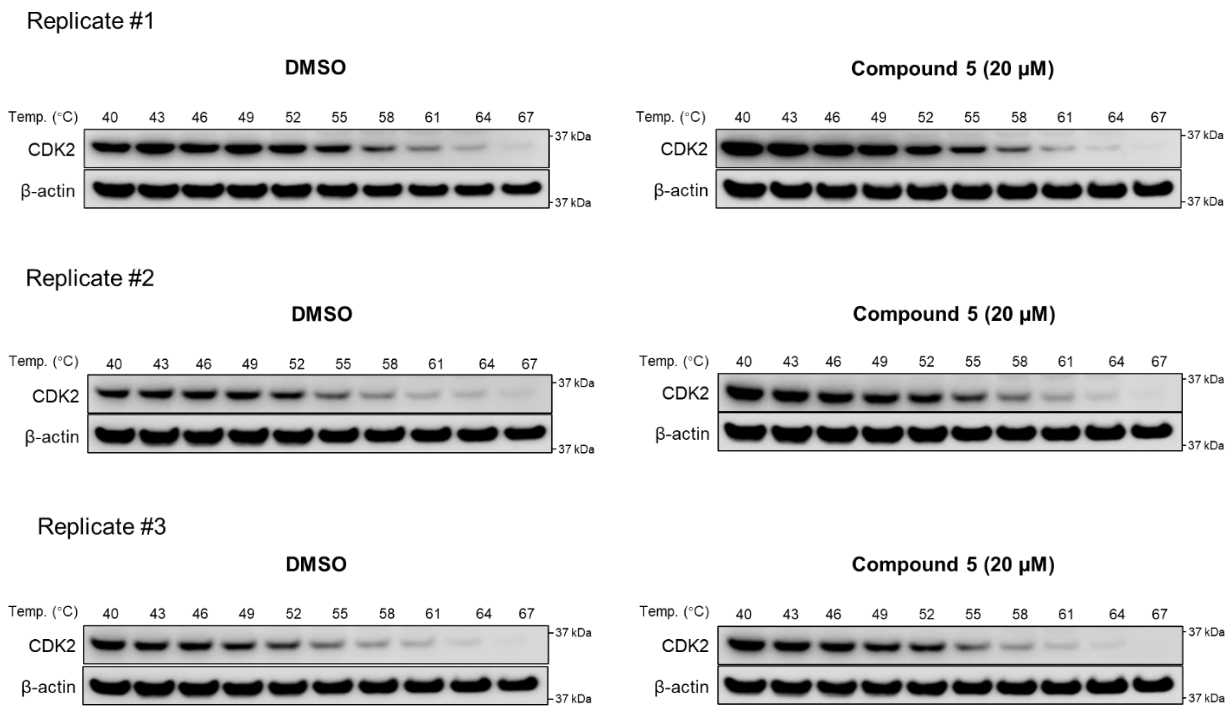

Supplementary Fig 14. **Triplicate CETSA data for DMSO (control) and compound 5 treated OVCAR-3 cells.** Numbers shown above bands on blots indicate the temperature of the experiment (°C). Source data are provided as a Source Data file.

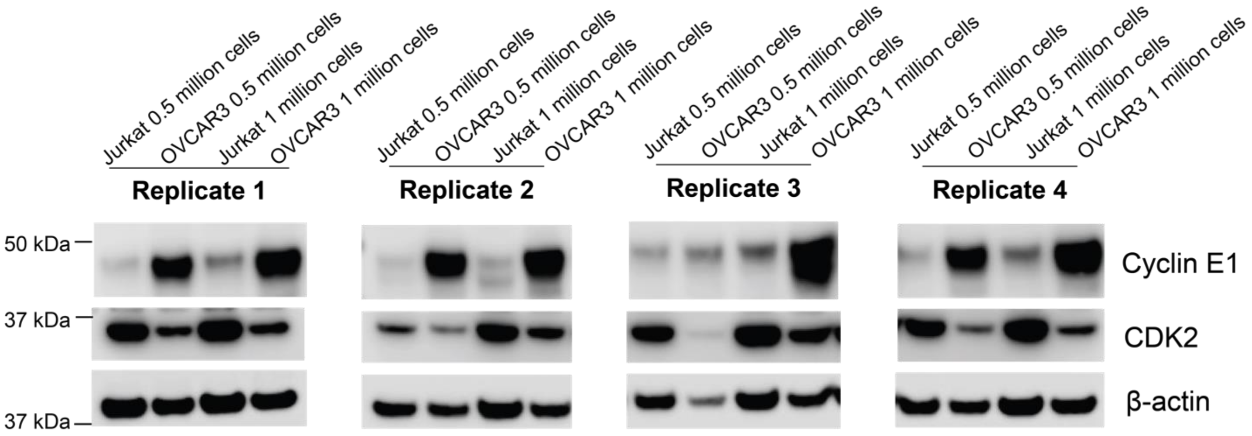

Supplementary Fig. 15. **Four replicates for cyclin E1 and CDK2 quantification experiments for Jurkat and OVCAR3 cells.** Source data are provided as a Source Data file.

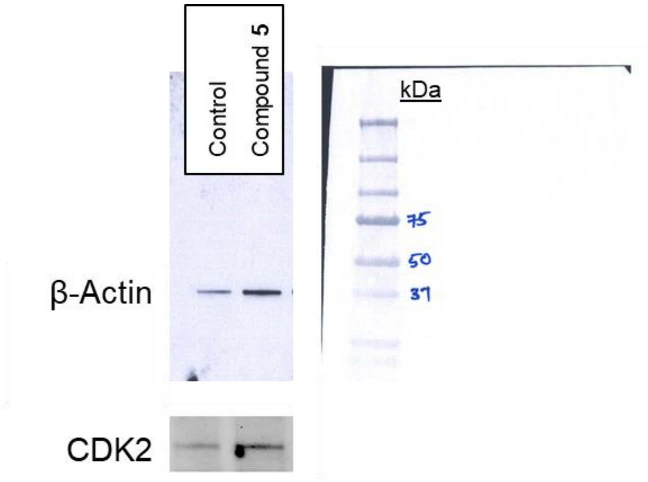

Supplementary Fig. 16. **Western blot showing robust expression of CDK2 in testis explants cultured in the presence of compound 5.** This analysis was performed once. Source data are provided as a Source Data file.

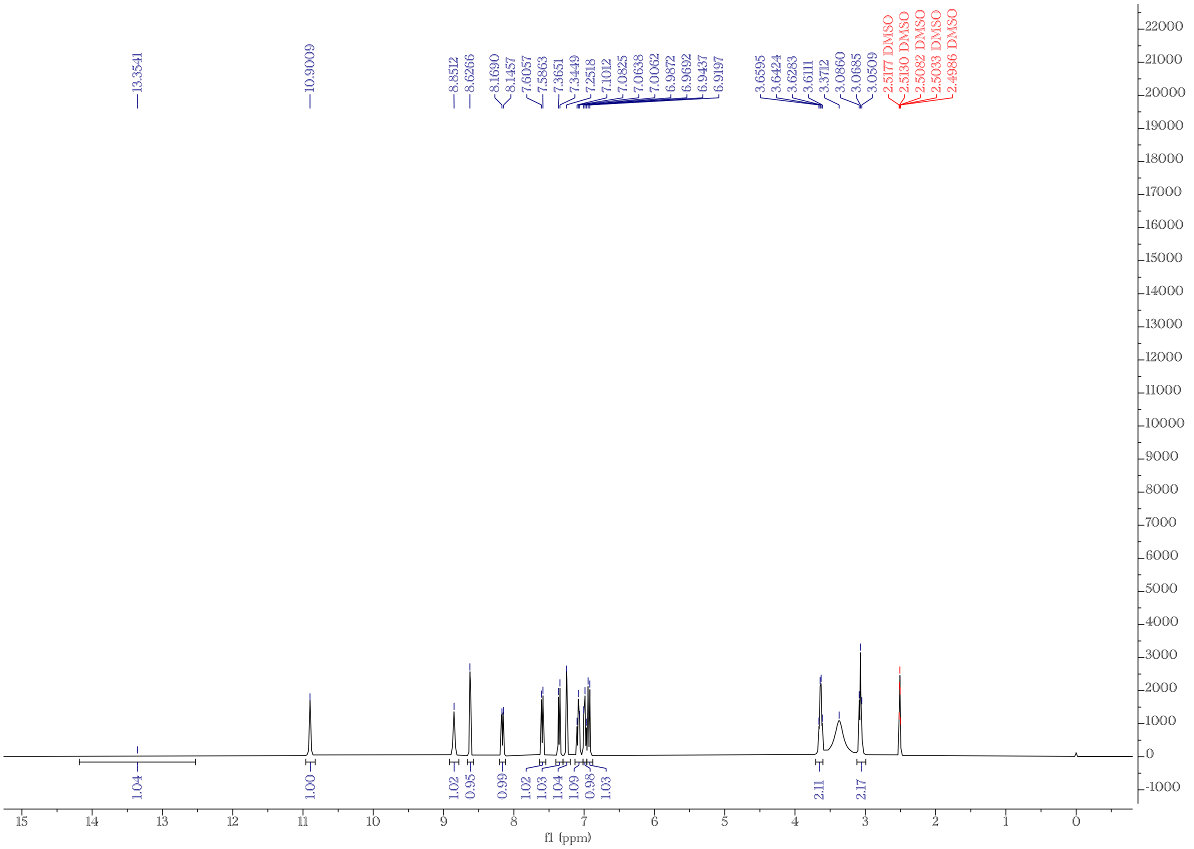

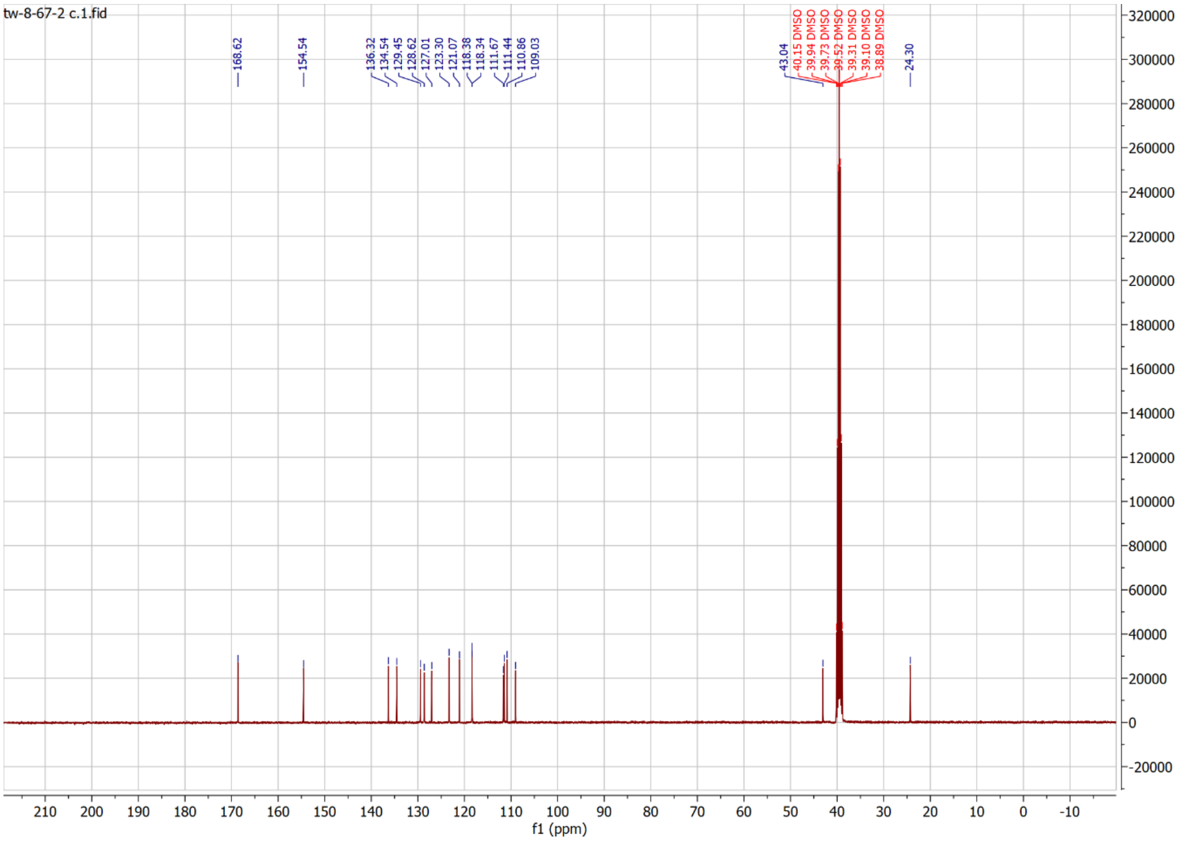

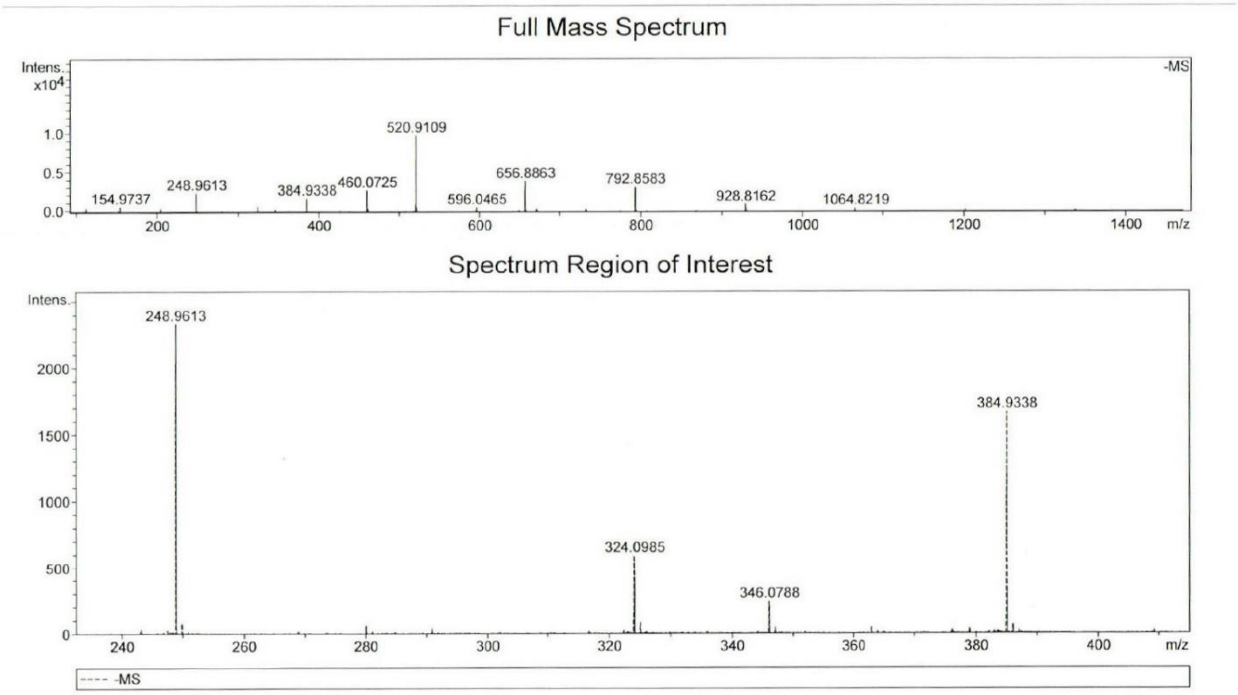

Supplementary Fig. 17. **^{1}H and ^{13}C NMR spectra and HRMS data of 1**.

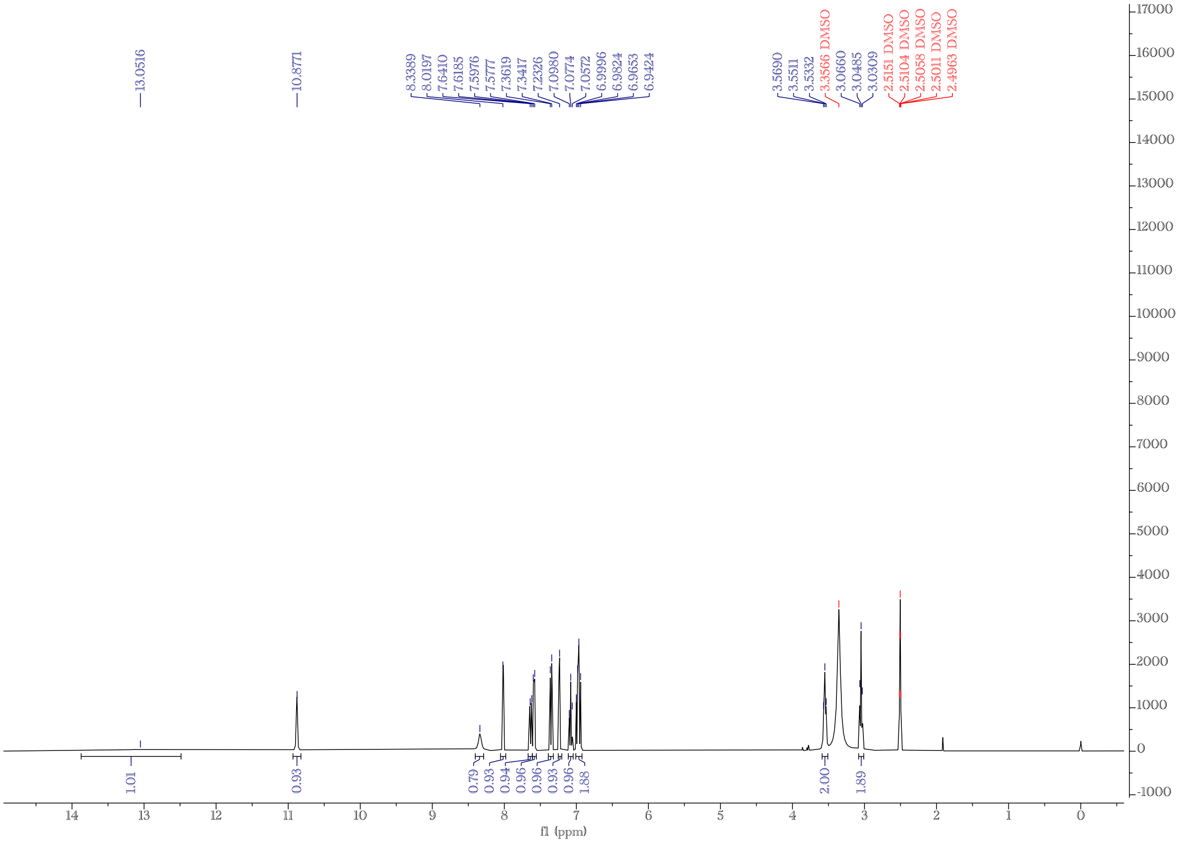
13.0516
10.8771
8.3389
8.0197
7.6410
7.6185
7.5976
7.5777
7.3619
7.3417
7.2326
7.0980
7.0774
7.0572
6.9996
6.9824
6.9653
6.9424
3.5690
3.5511
3.5332
3.3566 DMSO
3.0660
3.0485
3.0309
2.5151 DMSO
2.5104 DMSO
2.5058 DMSO
2.5011 DMSO
2.4963 DMSO
1.01
0.93
0.79
0.93
0.94
0.96
0.96
0.93
0.96
1.88
2.00
1.89
f1 (ppm)

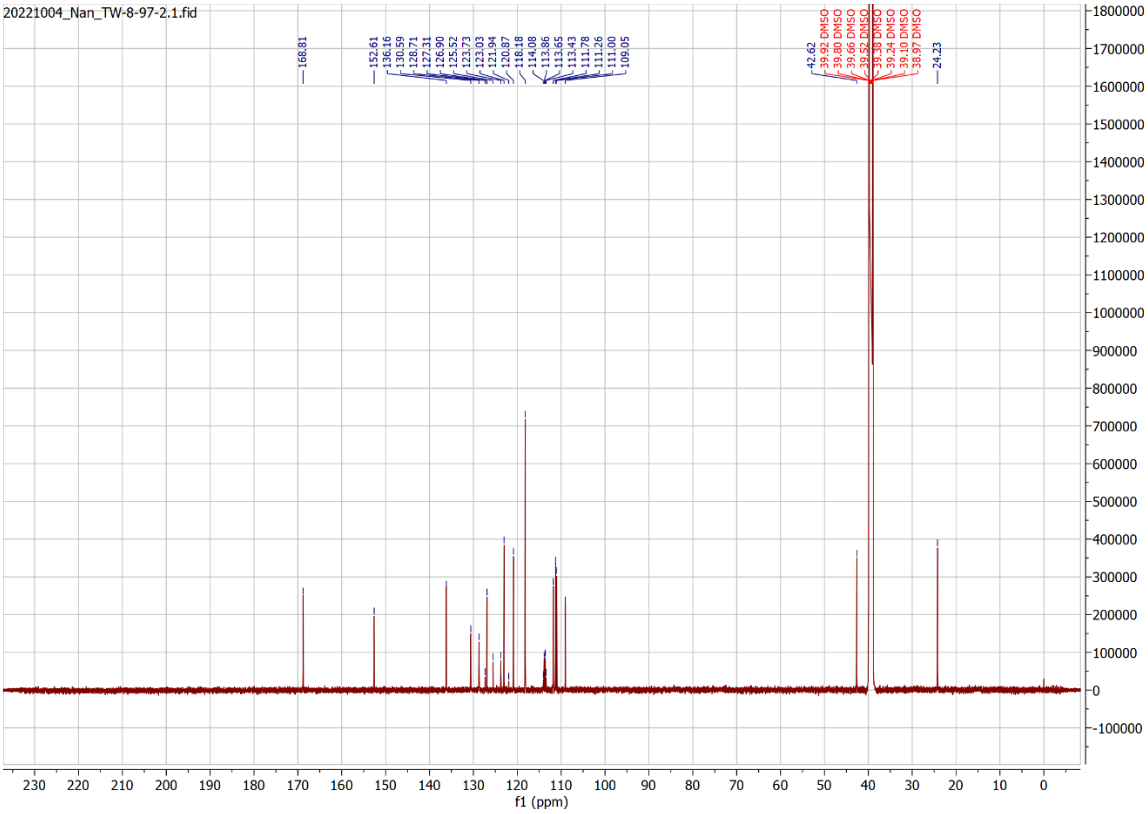
20221004_Nan_TW-8-97-2.1.fid
168.81
152.61
136.16
130.59
128.71
127.31
126.90
125.52
123.73
123.03
121.94
120.87
118.18
114.08
113.86
113.65
113.43
111.78
111.26
111.00
109.05
42.62
39.92 DMSO
39.80 DMSO
39.66 DMSO
39.52 DMSO
39.38 DMSO
39.24 DMSO
39.10 DMSO
38.97 DMSO
24.23
f1 (ppm)

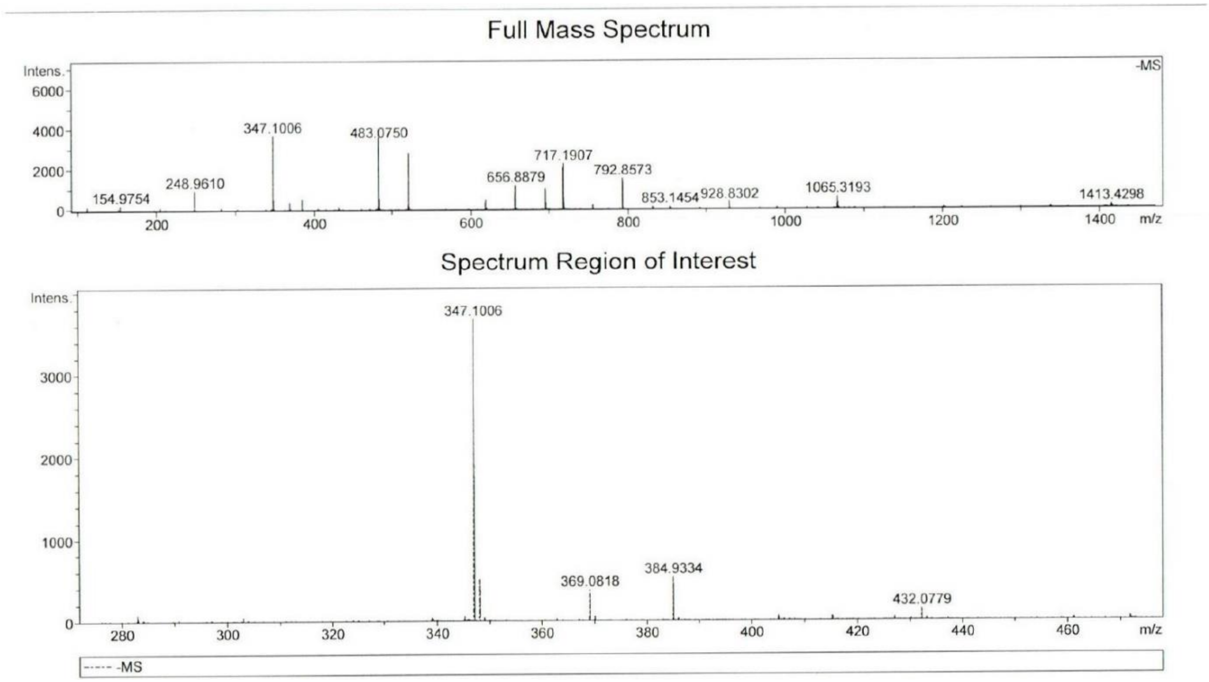

Supplementary Fig. 18. **^{1}H and ^{13}C NMR spectra and HRMS data of 2**.

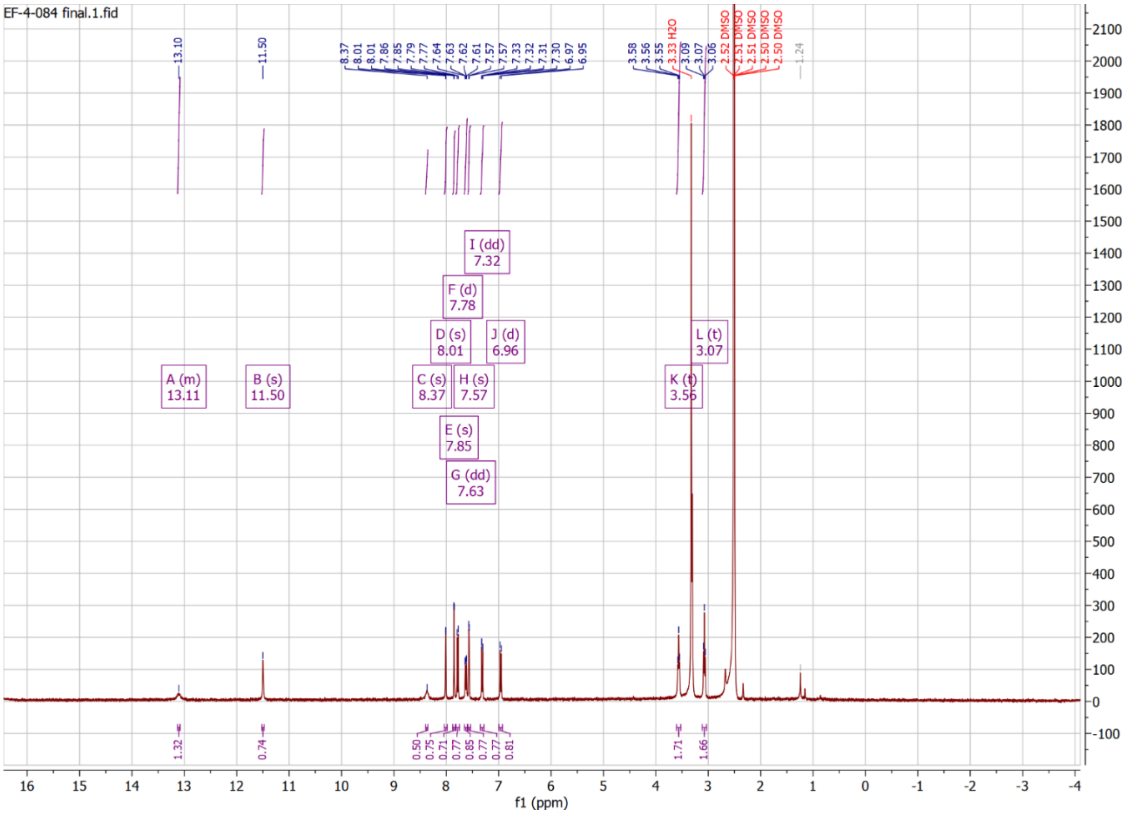

EF-4-084 final.1.fid
f1 (ppm)

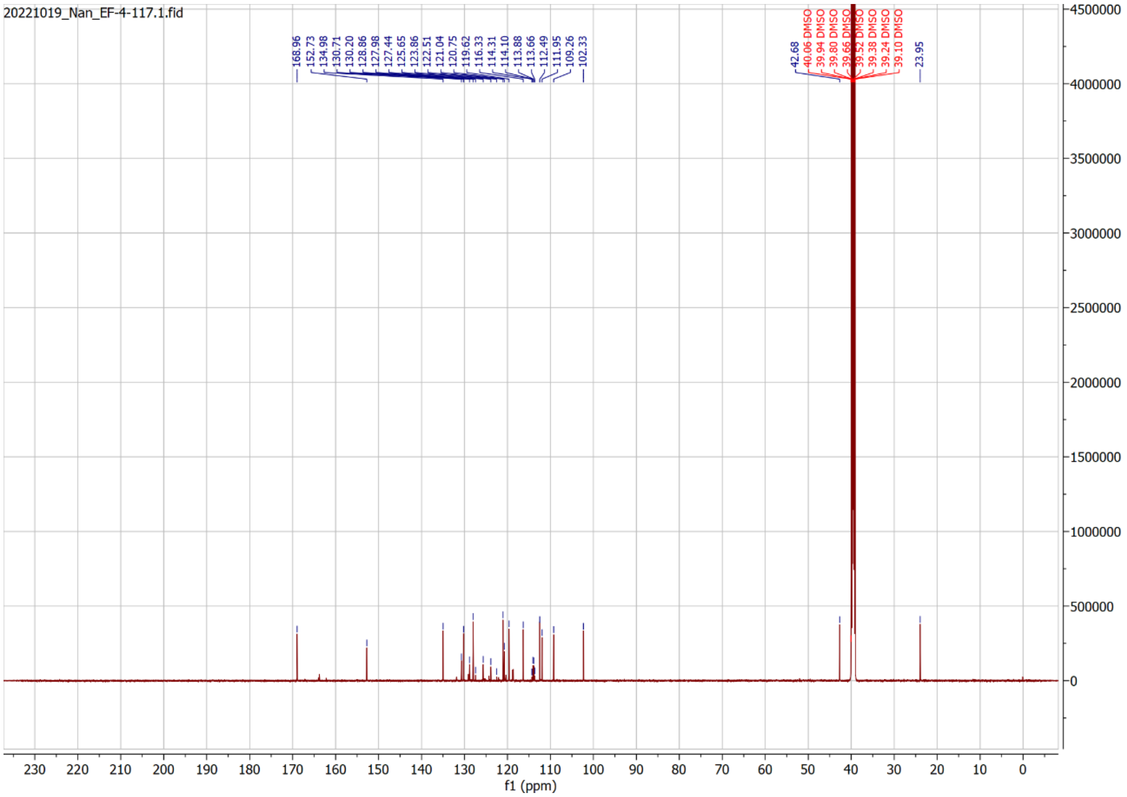

20221019_Nan_EF-4-117.1.fid
f1 (ppm)

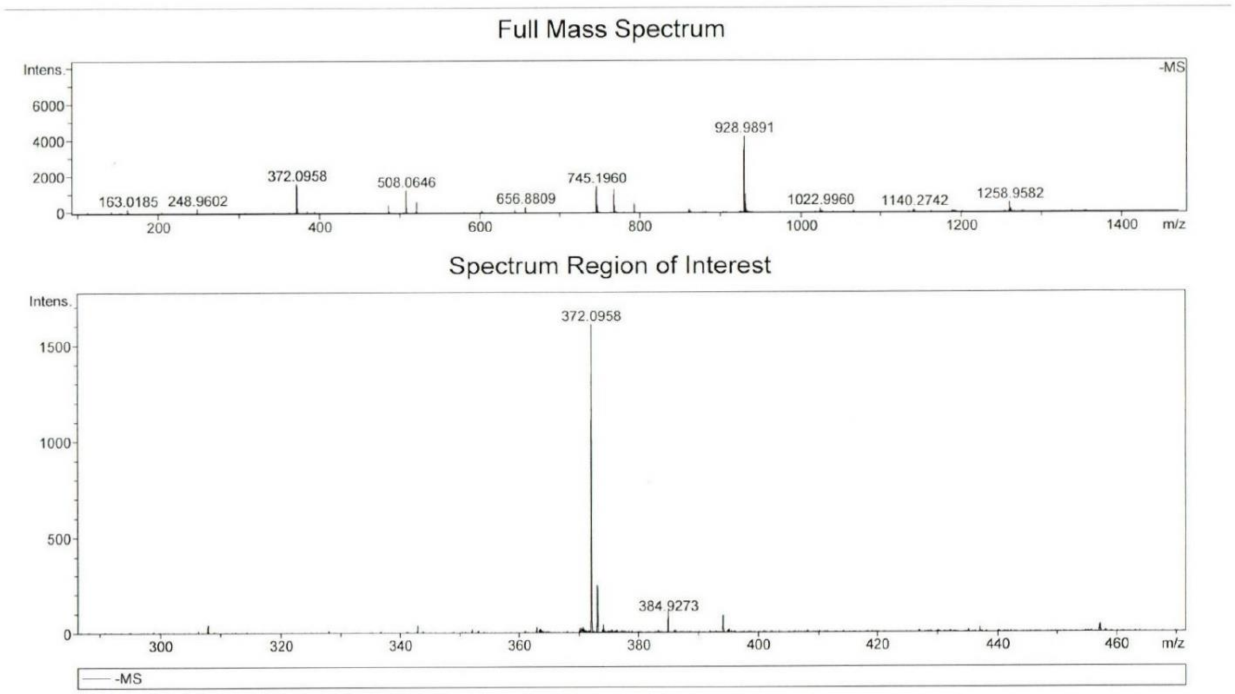

Supplementary Fig. 19. **^{1}H and ^{13}C NMR spectra and HRMS data of 3**.

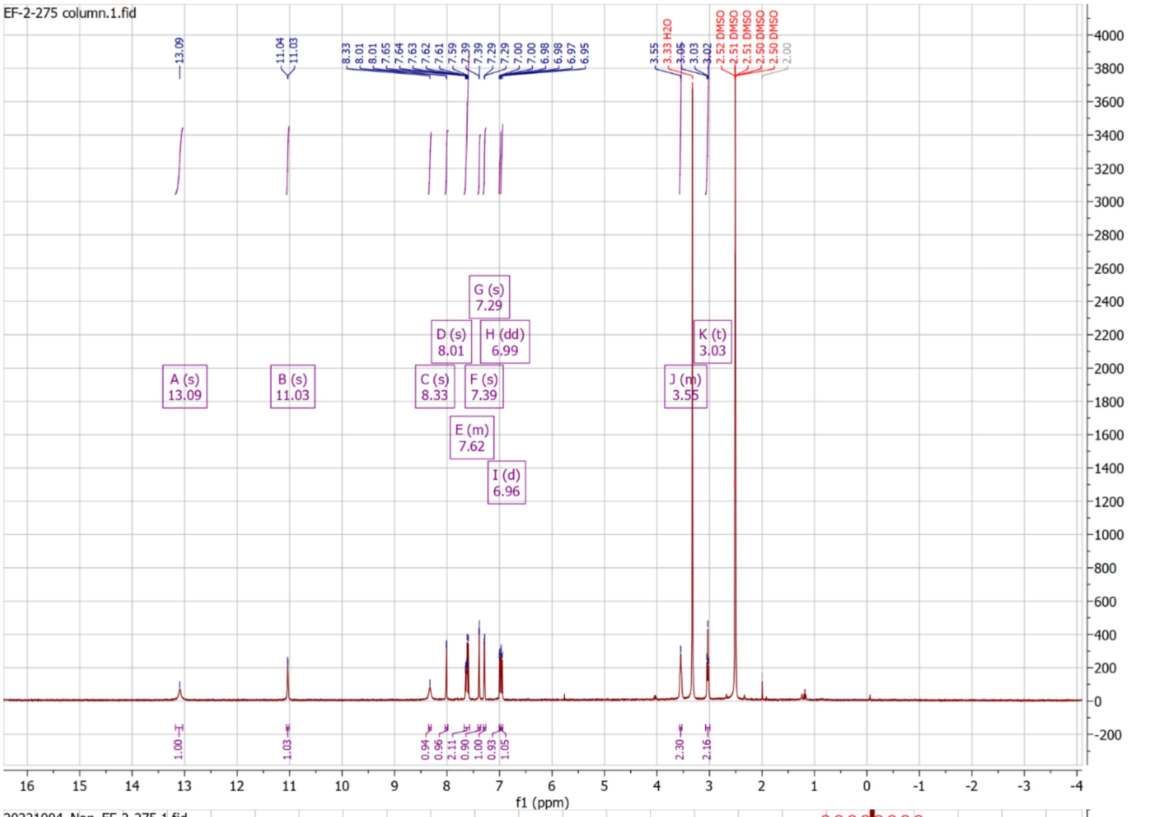
EF-2-275 column.1.fid
A (s) 13.09
B (s) 11.03
C (s) 8.33
D (s) 8.01
E (m) 7.62
F (s) 7.39
G (s) 7.29
H (dd) 6.99
I (d) 6.96
J (m) 3.55
K (t) 3.03
f1 (ppm)

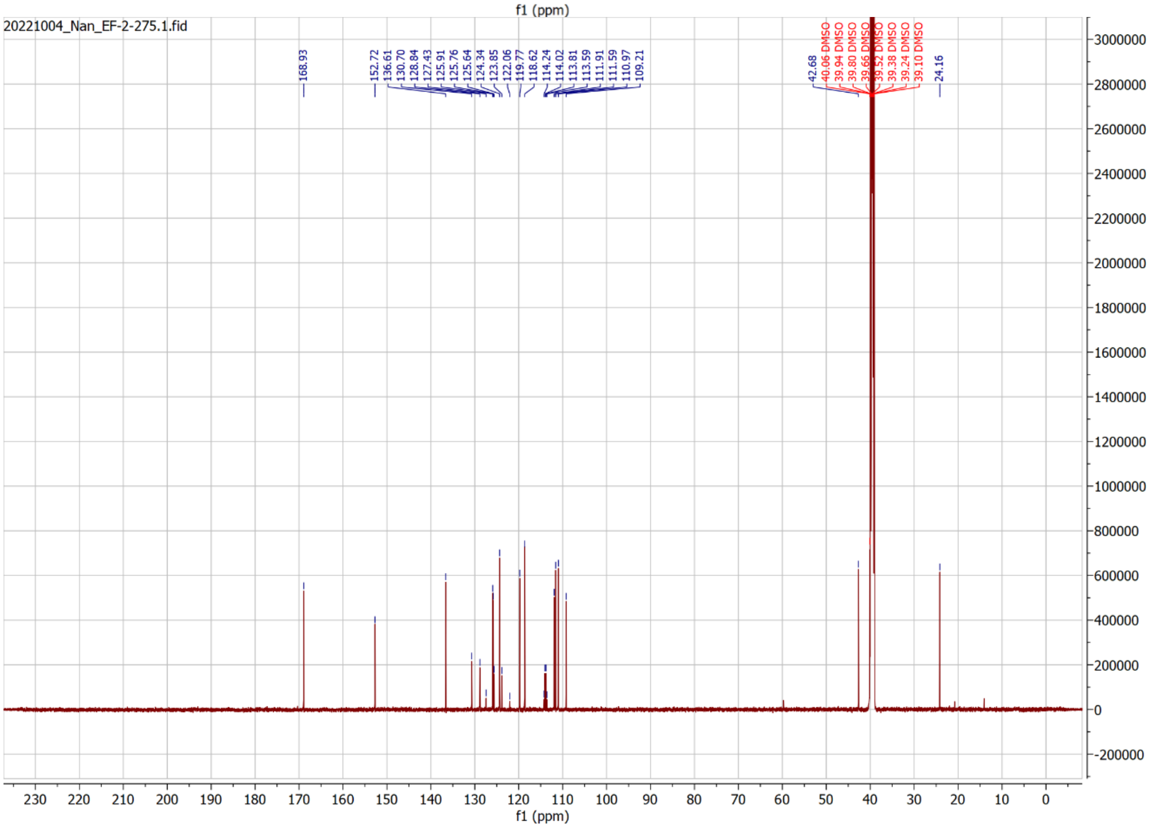
20221004_Nan_EF-2-275.1.fid
168.93
152.72
136.61
130.70
128.84
127.43
125.91
125.76
125.64
124.34
123.85
122.06
119.77
118.62
114.24
114.02
113.81
113.59
111.91
111.59
110.97
109.21
42.68
24.16
f1 (ppm)

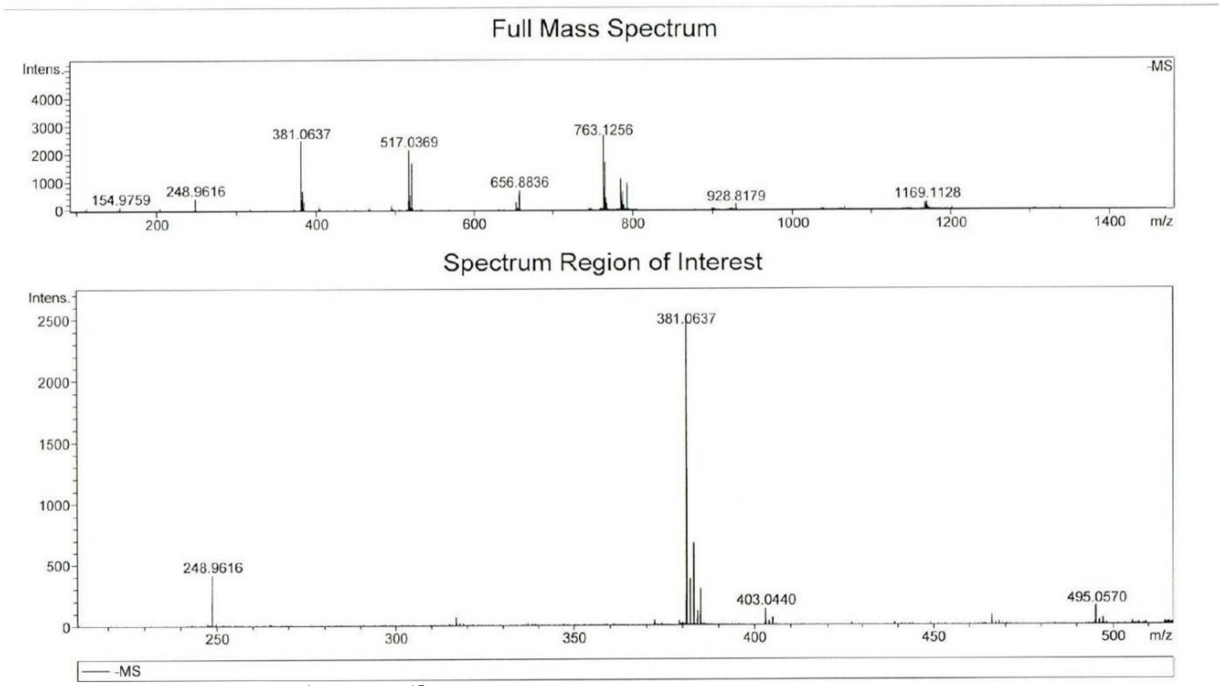

Supplementary Fig. 20. **^{1}H and ^{13}C NMR spectra and HRMS data of 4**.

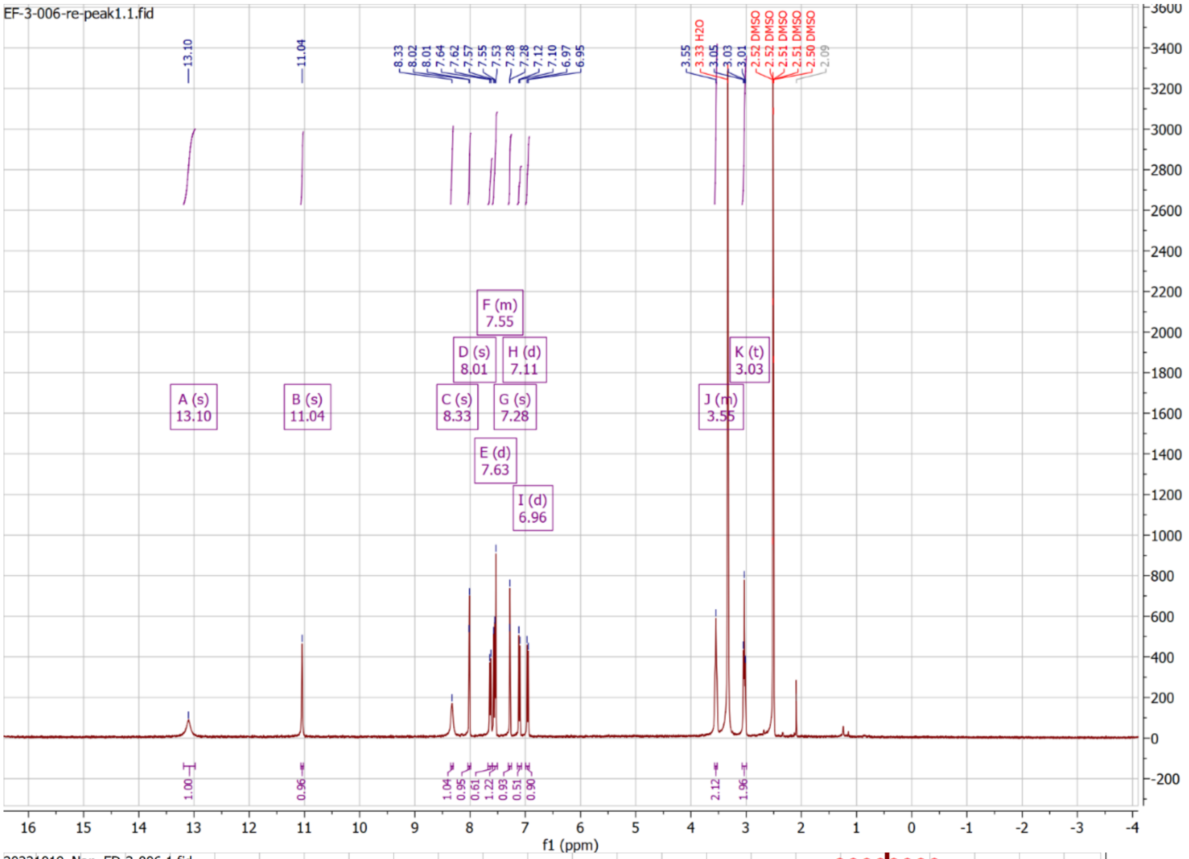
EF-3-006-re-peak1.1.fid
13.10
11.04
8.33 8.02 8.01 7.64 7.62 7.57 7.55 7.53 7.28 7.28 7.12 7.10 6.97 6.95
3.55 3.33 H2O 3.05 3.03 3.01 2.52 DMSO 2.52 DMSO 2.51 DMSO 2.51 DMSO 2.50 DMSO 2.09
A (s) 13.10
B (s) 11.04
C (s) 8.33
D (s) 8.01
E (d) 7.63
F (m) 7.55
G (s) 7.28
H (d) 7.11
I (d) 6.96
J (m) 3.55
K (t) 3.03
1.00
0.96
1.04 0.95 0.61 1.22 0.93 0.51 0.90
2.12
1.96
f1 (ppm)

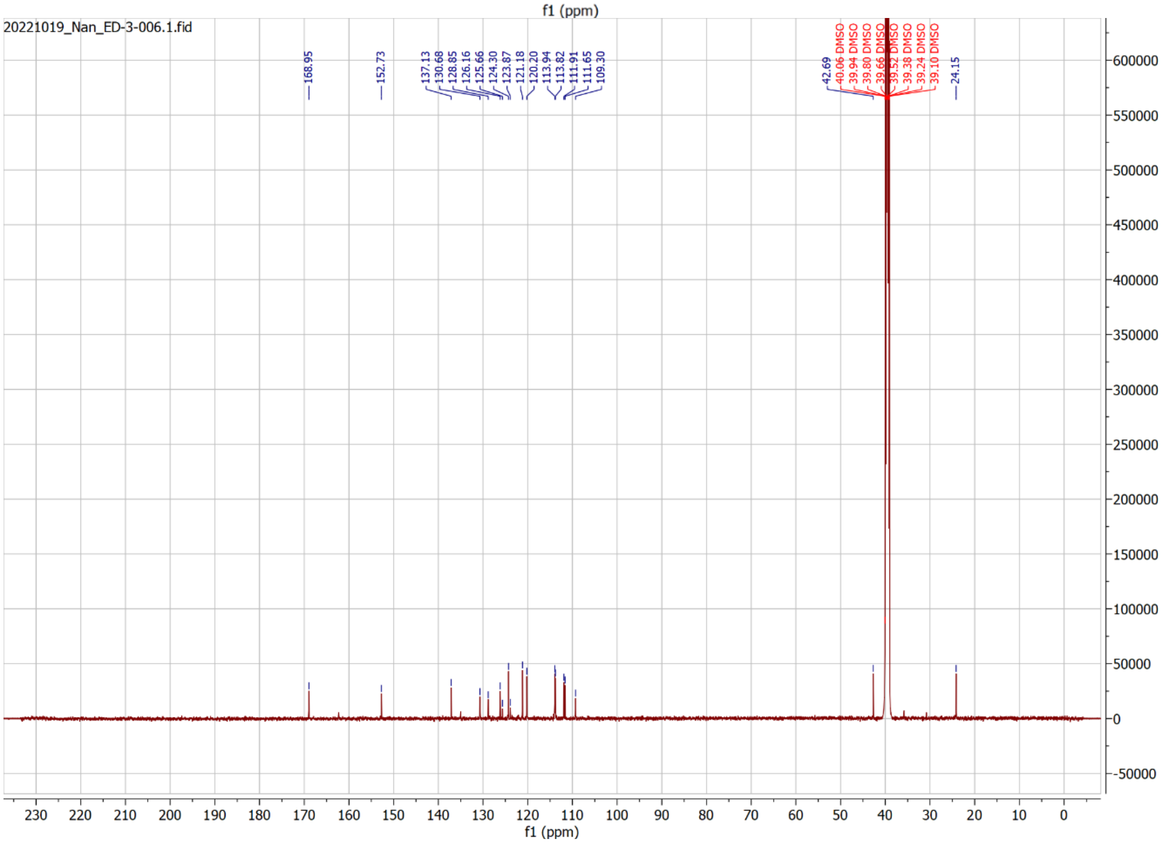
20221019_Nan_ED-3-006.1.fid
168.95
152.73
137.13 130.68 128.85 126.16 125.66 124.30 123.87 121.18 120.20 113.94 113.82 111.91 111.65 109.30
42.69 40.06 DMSO 39.94 DMSO 39.80 DMSO 39.66 DMSO 39.52 DMSO 39.38 DMSO 39.24 DMSO 39.10 DMSO
24.15
f1 (ppm)

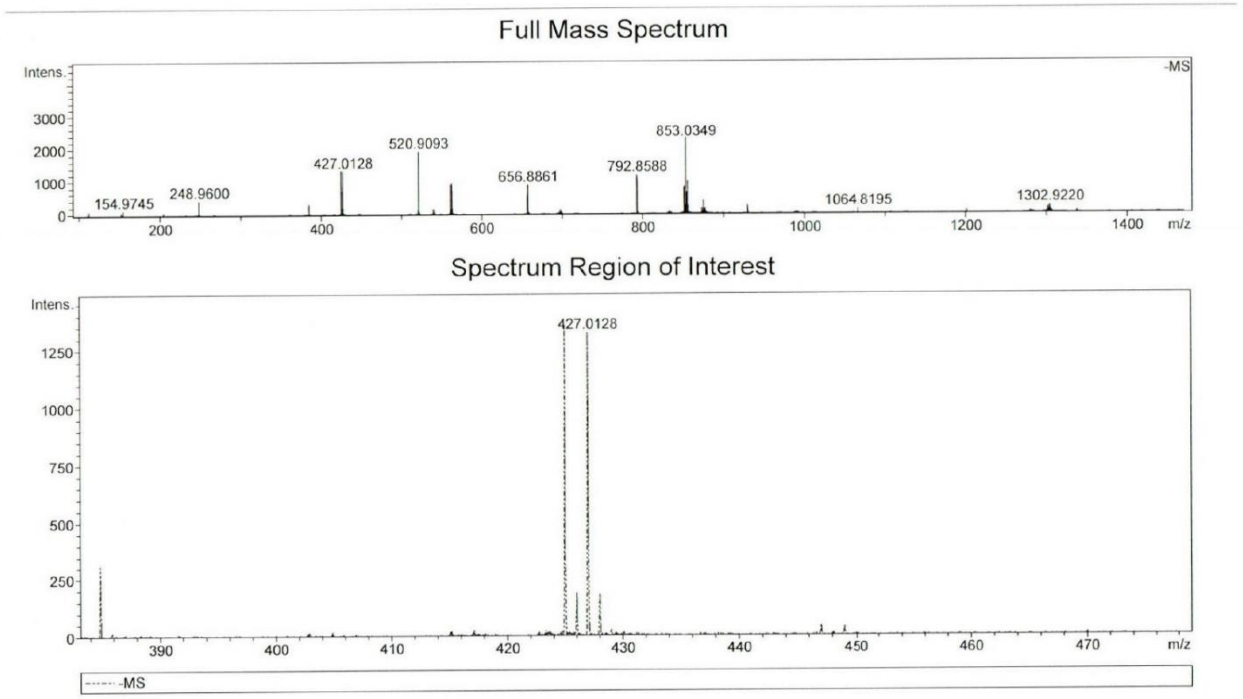

Supplementary Fig. 21. **^{1}H and ^{13}C NMR spectra and HRMS data of 5**.

Supplementary Table 1: Crystallographic data and refinement statistics

Protein		CDK2				
Inhibitor		Compound 1	Compound 2	Compound 1 & Dinaciclib	Compound 1 & Roscovitine	ATP
PDB ID		7RWF	7S84	8FOW	8FP0	8FP5
Ligand code		7TW	8IL	7TW + 1QK	7TW + RRC	ATP
Wavelength		1.54178	1.03318	1.033167	1.033167	1.54178
		Data reduction				
Space group		P 21 21 21	P 21 21 21	P 21 21 21	P 21 21 21	P 21 21 21
Unit cell dimensions	a	50.80	54.19	53.93	53.82	53.67
	b	70.24	71.55	71.81	72.10	71.71
	c	71.44	72.22	72.45	72.50	72.04
	α	90	90	90	90	90
	β	90	90	90	90	90
	γ	90	90	90	90	90
Resolution range (Å)		41.4 - 1.5 (1.554 - 1.5)	43.2 - 2.0 (2.072 - 2.0)	36.22 - 1.60 (1.64 - 1.60)	37.07 - 1.60 (1.64 - 1.60)	27.6 - 1.70 (1.74 - 1.70)
Unique reflections		42774 (4172)	19444 (1879)	35984 (2135)	37459 (2564)	31044 (2197)
Rmeas		0.113 (0.478)	0.119 (0.751)	0.066 (1.208)	0.045 (0.992)	0.035 (1.041)
CC(1/2)		0.994 (0.787)	0.998 (0.885)	0.998 (0.706)	1.0 (0.687)	1.0 (0.55)
Completeness (%)		99.9 (99.3)	99.4 (98.7)	95.2 (77.6)	98.7 (92.1)	99.3 (96.5)
I/σI		20.9 (2.0)	9.8 (2.1)	14.5 (1.63)	17.7 (1.14)	23.7 (1.32)
		Structure refinement				
Rwork (%)		19.22 (20.53)	19.69 (22.46)	20.4 (31.7)	21.1 (34.8)	19.4 (28.6)
Rfree[a] (%)		20.94 (21.81)	24.45 (30.87)	22.1 (39.6)	24.0 (36.6)	23.0 (33.8)
Wilson B (Å^2)		17.01	35.32	27.6	29.5	23.2
Average B (Å²)	all	21.10	35.13	38.1	42.6	32.4
	protein	20.78	31.38	38.1	42.4	32.4
	ligand	19.32	33.96	36.5	47.8	31.7
	solvent	26.09	38.81	39.0	42.4	33.4
rmsd[b] bond lengths (Å)		0.007	0.009	0.007	0.007	0.009
rmsd angles (deg)		0.93	1.04	1.1	0.94	1.18
Ramachandran	favored (%)	98.32	98.16	98.13	97.37	98.24
	allowed (%)	1.68	1.84	1.87	2.63	1.76
	outliers (%)	0	0	0	0	0

Values in paranthesis are for the highest resolution bins.
[a] Rfree is Rcryst calculated for randomly chosen unique reflections, which were excluded from the refinement.
[b] rmsd = root-mean-square deviation from ideal values.

Supplementary Table 2: Compound **4** does not substantially inhibit any CDK/cyclin complex at a concentration well above its K_D. Data provided by Reaction Biology in n=2 technical replicates.

Kinases	% Enzyme Activity (relative to DMSO controls) 1 µM compound 4	
	Rep 1	Rep 2
CDK1/cyclin A	95.2	93.7
CDK1/cyclin B	88.9	87.0
CDK2/cyclin A	98.4	95.1
CDK2/cyclin A1	99.8	99.4
CDK2/cyclin E	90.0	87.7
CDK2/cyclin O	95.7	95.0
CDK3/cyclin E	95.7	95.2
CDK4/cyclin D1	82.8	82.0
CDK5/P25	92.3	91.0
CDK6/cyclin D1	92.9	92.0
CDK7/cyclin H	97.6	94.9
CDK9/cyclin T1	98.4	97.1

Supplementary Table 3: Kinome screen of compound 5 at 10 μM concentration. Data provided by Reaction Biology.

Kinase	% Activity at 10 μM cmpd. 5 (mean ± s.d.)	Kinase	% Activity at 10 μM cmpd. 5 (mean ± s.d.)	Kinase	% Activity at 10 μM cmpd. 5 (mean ± s.d.)	Kinase	% Activity at 10 μM cmpd. 5 (mean ± s.d.)	Kinase	% Activity at 10 μM cmpd. 5 (mean ± s.d.)
ABL1	98.7 ± 0.5	CK1D	33.9 ± 2.4	HGK/MAP4K4	50.4 ± 0.4	MST3/STK24	103.7 ± 1.9	ROCK2	90.0 ± 2.0
ABL2/ARG	94.7 ± 2.8	CK1EPSILON	60.1 ± 1.8	HIPK1	64.9 ± 0.2	MST4	56.0 ± 0.2	RON/MST1R	89.7 ± 2.7
ACK1	92.0 ± 0.4	CK1G1	96.2 ± 0.3	HIPK2	79.9 ± 0.7	MUSK	104.4 ± 1.0	ROS/ROS1	94.7 ± 1.4
AKT1	93.4 ± 0.2	CK1G2	101.8 ± 1.6	HIPK3	86.3 ± 2.3	MYLK3	90.9 ± 3.2	RSK1	83.1 ± 2.8
AKT2	88.6 ± 2.8	CK1G3	96.3 ± 0.6	HIPK4	129.3 ± 6.2	MYLK4	78.7 ± 0.2	RSK2	94.3 ± 4.2
AKT3	90.2 ± 0.5	CK2A	59.1 ± 0.1	HPK1/MAP4K1	77.2 ± 0.7	MYO3A	118.0 ± 3.4	RSK3	87.0 ± 1.4
ALK	93.8 ± 0.7	CK2A2	46.9 ± 0.5	IGF1R	93.2 ± 0.1	MYO3B	97.8 ± 1.5	RSK4	91.0 ± 0.4
ALK1/ACVRL1	67.3 ± 1.4	CLK1	109.3 ± 0.4	IKKA/CHUK	83.2 ± 1.4	NEK1	91.8 ± 2.3	SBK1	83.7 ± 1.2
ALK2/ACVR1	74.1 ± 1.8	CLK2	104.3 ± 0.7	IKKb/IKBKB	82.1 ± 0.2	NEK11	87.7 ± 1.1	SGK1	90.5 ± 2.4
ALK3/BMPR1A	71.1 ± 2.0	CLK3	131.3 ± 0.6	IKKE/IKBKE	100.7 ± 3.0	NEK2	50.8 ± 1.3	SGK2	63.9 ± 2.0
ALK4/ACVR1B	66.7 ± 0.1	CLK4	97.4 ± 0.8	IR	64.7 ± 1.1	NEK3	93.3 ± 0.4	SGK3/SGKL	104.9 ± 0.9
ALK5/TGFBR1	84.2 ± 0.1	COT1/MAP3K8	88.4 ± 0.6	IRAK1	102.3 ± 2.5	NEK4	80.3 ± 3.4	SIK1	69.3 ± 1.0
ALK6/BMPR1B	96.7 ± 0.2	CSK	100.9 ± 0.9	IRAK2	145.0 ± 0.9	NEK5	94.7 ± 0.5	SIK2	81.0 ± 0.3
ARAF	91.1 ± 0.8	CTK/MATK	71.1 ± 3.8	IRAK4	115.1 ± 0.2	NEK6	77.6 ± 0.1	SIK3	64.3 ± 0.5
ARK5/NUAK1	75.5 ± 0.1	DAPK1	108.8 ± 1.5	IRR/INSRR	76.1 ± 4.0	NEK7	80.2 ± 0.8	SLK/STK2	89.7 ± 3.0
ASK1/MAP3K5	110.3 ± 2.8	DAPK2	78.8 ± 0.4	ITK	127.0 ± 0.1	NEK9	117.4 ± 3.3	SNARK/NUAK2	87.7 ± 2.3
AURORA A	75.2 ± 1.8	DCAMKL1	96.0 ± 3.9	JAK1	67.7 ± 0.8	NIM1	88.9 ± 1.0	SNRK	80.4 ± 2.1
AURORA B	34.9 ± 0.5	DCAMKL2	101.3 ± 0.6	JAK2	78.8 ± 1.9	NLK	81.4 ± 4.1	SRMS	86.4 ± 1.3
AURORA C	98.0 ± 0.2	DDR1	87.7 ± 0.4	JAK3	94.7 ± 0.1	OSR1/OXSR1	59.4 ± 2.8	SRPK1	91.3 ± 0.5
AXL	67.9 ± 1.8	DDR2	103.2 ± 3.6	JNK1	88.4 ± 1.1	P38A/MAPK14	124.8 ± 0.6	SRPK2	95.1 ± 0.0
BLK	92.7 ± 1.3	DMPK	108.2 ± 0.2	JNK2	89.6 ± 0.4	P38B/MAPK11	140.1 ± 2.3	SSTK/TSSK6	101.9 ± 3.0
BMPR2	88.0 ± 5.4	DMPK2	96.2 ± 1.7	JNK3	90.9 ± 0.3	P38D/MAPK13	80.9 ± 2.2	STK16	80.4 ± 1.1
BMX/ETK	101.8 ± 0.4	DRAK1/STK17A	108.8 ± 2.6	KDR/VEGFR2	87.7 ± 1.6	P38G	106.6 ± 1.5	STK21/CIT	102.1 ± 0.3
BRAF	98.7 ± 0.5	DYRK1/DYRK1A	80.0 ± 5.9	KHS/MAP4K5	72.0 ± 2.8	P70S6K/RPS6KB1	85.4 ± 0.7	STK22D/TSSK1	103.5 ± 0.1
BRK	107.9 ± 2.4	DYRK1B	64.8 ± 1.1	KSR1	92.9 ± 2.5	P70S6KB/RPS6KB2	96.1 ± 1.6	STK25/YSK1	123.6 ± 1.4
BRSK1	77.2 ± 2.3	DYRK3	57.3 ± 2.4	KSR2	83.3 ± 1.5	PAK1	86.8 ± 0.9	STK32B/YANK2	154.6 ± 0.7
BRSK2	79.5 ± 0.8	DYRK4	96.9 ± 2.5	LATS1	93.0 ± 3.5	PAK2	95.6 ± 0.3	STK32C/YANK3	100.2 ± 0.6
BTK	89.2 ± 0.1	EGFR	87.8 ± 1.0	LATS2	79.1 ± 1.6	PAK3	84.0 ± 0.9	STK33	85.1 ± 4.1
C-KIT	84.5 ± 2.5	EPHA1	102.2 ± 0.1	LCK	76.5 ± 2.7	PAK4	99.7 ± 2.2	STK38/NDR1	88.0 ± 0.7
C-MER	52.1 ± 1.5	EPHA2	93.1 ± 0.5	LCK2/ICK	98.2 ± 1.3	PAK5	101.2 ± 0.3	STK38L/NDR2	94.0 ± 0.9
C-MET	108.8 ± 4.5	EPHA3	99.7 ± 1.6	LIMK1	84.2 ± 0.0	PAK6	243.5 ± 9.0	STK39/STLK3	113.4 ± 0.1
C-SRC	95.1 ± 0.2	EPHA4	94.3 ± 0.5	LIMK2	75.1 ± 1.7	PASK	95.2 ± 2.9	SYK	85.8 ± 1.6
CAMK1A	69.1 ± 1.0	EPHA5	93.4 ± 0.3	LKB1	93.7 ± 1.8	PBK/TOPK	156.1 ± 1.8	TAK1	92.1 ± 0.7
CAMK1B	59.1 ± 0.7	EPHA6	91.9 ± 1.1	LOK/STK10	88.1 ± 0.3	PDGFRA	86.9 ± 0.2	TAOK1	134.4 ± 0.7
CAMK1D	69.8 ± 1.7	EPHA7	82.8 ± 2.2	LRRK2	99.5 ± 0.1	PDGFRB	92.5 ± 0.1	TAOK2/TAO1	100.9 ± 0.2
CAMK1G	40.8 ± 2.3	EPHA8	100.5 ± 2.7	LYN	99.5 ± 2.0	PDK1/PDPK1	97.0 ± 1.0	TAOK3/JIK	83.3 ± 2.1
CAMK2A	70.2 ± 3.3	EPHB1	91.2 ± 0.0	LYN B	93.2 ± 0.3	PHKG1	87.0 ± 2.6	TBK1	117.1 ± 1.2
CAMK2B	62.2 ± 0.8	EPHB2	89.8 ± 0.0	MAK	81.6 ± 0.5	PHKG2	106.5 ± 0.1	TEC	100.8 ± 1.6
CAMK2D	78.3 ± 3.4	EPHB3	84.2 ± 0.7	MAPKAPK2	95.2 ± 1.8	PIM1	40.6 ± 3.2	TESK1	82.4 ± 1.3
CAMK2g	74.4 ± 0.0	EPHB4	91.7 ± 0.5	MAPKAPK3	92.8 ± 0.2	PIM2	92.4 ± 0.6	TESK2	78.7 ± 0.8
CAMK4	27.5 ± 6.5	ERBB2/HER2	78.0 ± 0.2	MAPKAPK5/PRAK	95.4 ± 2.0	PIM3	20.6 ± 1.0	TGFBR2	79.2 ± 1.5
CAMKK1	65.6 ± 1.3	ERBB4/HER4	96.2 ± 1.0	MARK1	93.3 ± 0.5	PKA	102.6 ± 3.5	TIE2/TEK	93.7 ± 0.9
CAMKK2	82.9 ± 0.9	ERK1	99.7 ± 0.9	MARK2/PAR-1BA	87.5 ± 0.7	PKACB	93.4 ± 0.4	TLK1	89.2 ± 1.6
CDC7/DBF4	77.9 ± 2.9	ERK2/MAPK1	117.9 ± 3.0	MARK3	82.7 ± 2.2	PKACG	76.2 ± 2.6	TLK2	99.4 ± 3.0
CDK1/cyclin A	95.4 ± 0.8	ERK5/MAPK7	120.8 ± 3.0	MARK4	69.6 ± 1.3	PKCA	123.2 ± 5.3	TNIK	51.9 ± 1.4
CDK1/CYCLIN B	31.0 ± 0.3	ERK7/MAPK15	135.7 ± 6.1	MAST3	94.9 ± 0.2	PKCB1	91.0 ± 0.0	TNK1	94.4 ± 0.5
CDK1/CYCLIN E	60.2 ± 0.3	ERN1/IRE1	101.0 ± 0.6	MASTL	101.7 ± 0.2	PKCB2	100.1 ± 1.4	TRKA	65.1 ± 1.6
CDK14/CYCLIN Y (PFTK1)	114.1 ± 1.5	ERN2/IRE2	49.1 ± 2.5	MEK1	103.7 ± 2.4	PKCD	54.7 ± 2.7	TRKB	45.4 ± 7.4
CDK15/CYCLIN A2	81.7 ± 1.4	FAK/PTK2	88.2 ± 1.5	MEK2	110.4 ± 0.1	PKCEPSILON	96.1 ± 0.2	TRKC	91.0 ± 0.5
CDK16/CYCLIN Y (PCTAIRE)	76.9 ± 0.5	FER	89.6 ± 2.0	MEK3	92.9 ± 2.3	PKCETA	130.9 ± 2.7	TSSK2	79.9 ± 2.4
CDK17/CYCLIN Y (PCTK2)	124.2 ± 0.0	FES/FPS	96.5 ± 1.8	MEK5	79.2 ± 0.8	PKCG	81.9 ± 1.6	TSSK3/STK22C	68.9 ± 0.2
CDK18/CYCLIN Y (PCTK3)	99.0 ± 0.1	FGFR1	102.6 ± 0.3	MEKK1	110.0 ± 0.1	PKCIOTA	96.8 ± 0.8	TTBK1	102.7 ± 3.1
CDK2/CYCLIN A	102.0 ± 1.7	FGFR2	101.3 ± 0.5	MEKK2	107.9 ± 0.2	PKCMU/PRKD1	115.9 ± 0.9	TTBK2	105.1 ± 1.1
CDK2/CYCLIN A1	94.7 ± 0.3	FGFR3	90.7 ± 1.5	MEKK3	106.6 ± 1.1	PKCNU/PRKD3	139.2 ± 7.1	TXK	80.5 ± 0.4
CDK2/CYCLIN E	85.6 ± 0.2	FGFR4	63.4 ± 6.7	MEKK6	107.9 ± 1.4	PKCTHETA	95.5 ± 0.4	TYK1/LTK	86.2 ± 1.1
CDK2/CYCLIN E2	92.6 ± 0.3	FGR	83.5 ± 1.1	MELK	95.8 ± 3.0	PKCZETA	126.6 ± 0.3	TYK2	78.1 ± 0.7
CDK2/CYCLIN O	83.1 ± 3.4	FLT1/VEGFR1	85.0 ± 0.7	MINK/MINK1	86.9 ± 0.4	PKD2/PRKD2	84.1 ± 0.4	TYRO3/SKY	95.9 ± 0.0
CDK3/CYCLIN E	89.0 ± 1.5	FLT3	95.2 ± 1.6	MKK4	114.7 ± 4.5	PKG1A	100.9 ± 1.8	ULK1	106.8 ± 0.3
CDK3/CYCLIN E2	85.1 ± 2.0	FLT4/VEGFR3	102.1 ± 3.7	MKK6	101.0 ± 0.2	PKG1B	78.0 ± 0.4	ULK2	102.4 ± 0.4
CDK4/CYCLIN D1	80.1 ± 2.6	FMS	98.0 ± 1.7	MKK7	90.6 ± 0.3	PKG2/PRKG2	117.2 ± 7.9	ULK3	89.9 ± 1.4
CDK4/CYCLIN D2	29.1 ± 0.1	FRK/PTK5	90.1 ± 1.1	MLCK/MYLK	57.6 ± 2.0	PKN1/PRK1	122.8 ± 1.9	VRK1	104.7 ± 1.5
CDK4/CYCLIN D3	82.9 ± 0.6	FYN	86.4 ± 1.8	MLCK2/MYLK2	70.7 ± 0.3	PKN2/PRK2	110.5 ± 1.7	VRK2	94.6 ± 1.0
CDK5/P25	48.6 ± 1.3	GCK/MAP4K2	97.4 ± 2.0	MLK1/MAP3K9	95.8 ± 0.2	PKN3/PRK3	98.3 ± 1.2	WNK1	99.7 ± 0.2
CDK5/P35	88.8 ± 0.3	GLK/MAP4K3	97.8 ± 0.3	MLK2/MAP3K10	74.8 ± 2.2	PLK1	92.7 ± 1.5	WNK2	109.6 ± 0.2
CDK6/CYCLIN D1	87.9 ± 0.1	GRK1	97.5 ± 0.3	MLK3/MAP3K11	68.1 ± 0.8	PLK2	67.4 ± 0.7	WNK3	100.7 ± 0.4
CDK6/CYCLIN D2	44.6 ± 0.5	GRK2	96.5 ± 0.4	MLK4	88.2 ± 0.3	PLK3	79.3 ± 3.7	YES/YES1	96.9 ± 1.3
CDK6/CYCLIN D3	60.9 ± 1.2	GRK3	93.7 ± 1.2	MNK1	79.0 ± 0.9	PLK4/SAK	91.2 ± 1.2	YSK4/MAP3K19	110.3 ± 0.7
CDK7/CYCLIN H	103.6 ± 3.6	GRK4	80.2 ± 0.2	MNK2	93.3 ± 1.9	PRKX	92.6 ± 1.1	ZAK/MLTK	96.9 ± 0.2
CDK9/CYCLIN K	88.6 ± 0.7	GRK5	100.8 ± 1.1	MRCKA/CDC42BPA	98.2 ± 0.3	PYK2	75.6 ± 1.9	ZAP70	95.1 ± 1.6
CDK9/CYCLIN T1	81.5 ± 1.5	GRK6	111.5 ± 2.8	MRCKB/CDC42BPB	99.6 ± 2.7	RAF1	97.8 ± 0.2	ZIPK/DAPK3	74.5 ± 3.3
CDK9/CYCLIN T2	84.5 ± 0.4	GRK7	101.5 ± 0.4	MSK1/RPS6KA5	82.2 ± 0.5	RET	92.7 ± 3.3		
CHK1	95.2 ± 1.2	GSK3a	67.6 ± 0.3	MSK2/RPS6KA4	32.1 ± 1.4	RIPK2	109.3 ± 2.9		
CHK2	90.2 ± 1.5	GSK3B	79.3 ± 7.6	MSSK1/STK23	69.7 ± 3.0	RIPK4	45.4 ± 0.9		
CK1A1	91.2 ± 0.3	HASPIN	59.9 ± 1.3	MST1/STK4	89.6 ± 2.7	RIPK5	35.9 ± 1.6		
CK1A1L	90.8 ± 1.1	HCK	86.2 ± 0.4	MST2/STK3	134.0 ± 0.4	ROCK1	93.3 ± 1.1		

Absolute qNMR data

(1) Sample preparation

Compounds and dimethyl sulfone (*TCI*, 100% by GC, lot VOA5G-OD) were weighed into 3 mm standard NMR tubes using an analytical balance (Mettler Toledo XS205) with 0.01 mg accuracy, then 170 uL of Acetone-d_6 was added into NMR tubes. All NMR tubes were capped and wrapped with PTFE tape and subsequently with paraffin tape. The compounds and the internal calibrant were dissolved completely before submitted to NMR instrument.

Compounds ID	Weight of compounds (mg)	Weight of $DMSO_2$ (mg)
**1**	5.70	1.65
**2**	2.44	4.01
**3**	4.07	2.62
**4**	2.12	1.91
**5**	2.81	2.28

(2) Acquisition

The instrument and controlled parameters used during the acquisition are listed below:

Pulse Program: Single pulse, without carbon decoupling ('s2pul' [Agilent/Varian]; 'zg' with 90° pulse [Bruker]; "single pulse" [Jeol])
Sample Temperature: 25 °C
Data Points: 64 K
Zero-Filling: to 256 K
Dummy Scans: 4

The table summarizes the used conditions for scans:

Pulse Width	$\mathbf{10^{o}}$
	RT
Relaxation delay	0
Acquisition time	4s
Spectral Window	30 ppm
Transmitter Offset	7.5 ppm
Number of Scans for 400 MHz	512

(3) Post-acquisition processing

Apodization: LB = 0.1 Hz
Zero-filling: 256 K
Phasing: Manual phase correction
Baseline correction: Bernstein Polynomials

(4) Quantitative measurement of integrals

The proton integrals and range (ppm) of all signals used for quantification have been documented below. The integral of the internal calibrant resonance signal (dimethyl sulfone) is in bold.

**1**	
Range	Absolute
7.06-7.00	1.02
6.98-6.93	1.01
3.77-3.70	2.03
3.22-3.16	2.00
**2.99-2.94**	**6.08**
$\boldsymbol{Int_t}$ = 1.01, $\boldsymbol{n_t}$ = 1<br>$\boldsymbol{MW_t}$ = 325.32 g/mol	

**2**	
Range	Absolute
7.30-7.26	0.99
3.14-3.08	1.05
3.69-3.63	2.00
3.21-3.15	2.00
**3.00-2.93**	**37.09**
$\boldsymbol{Int_t}$ = 1.01, $\boldsymbol{n_t}$ = 1<br>$\boldsymbol{MW_t}$ = 348.33 g/mol	

**3**	
Range	Absolute
7.35-7.30	1.01
7.04-7.00	1.01
3.71-3.65	2.00
3.25-3.18	2.05
**3.00-2.95**	**15.51**
$\boldsymbol{Int_t}$ = 1.01, $\boldsymbol{n_t}$ = 1<br>$\boldsymbol{MW_t}$ = 373.34 g/mol	

**4**	
Range	Absolute
7.35-7.31	1.00
7.06-6.98	1.99
3.70-3.62	2.00
3.20-3.13	2.21
**3.01-2.94**	**45.48**
$\boldsymbol{Int_t}$ = 1.02, $\boldsymbol{n_t}$ = 1<br>$\boldsymbol{MW_t}$ = 382.77 g/mol	

5	
Range	Absolute
7.19-7.13	1.01
7.03-6.97	1.02
3.69-3.62	2.00
3.19-3.13	2.09
**3.00-2.95**	**22.52**
$\boldsymbol{Int_t}$ = 1.02, $\boldsymbol{n_t}$ = 1<br>$\boldsymbol{MW_t}$ = 426.02 g/mol	

(5) Calculation

$$P\% = \frac{n_{IC} \times Int_t \times MW_t \times m_{IC}}{n_t \times Int_{IC} \times MW_{IC} \times m_s} \times P_{IC}$$

- P – purity of the target analyte, %
- P_{IC} – purity of the internal calibrant, %
- n_{IC} – number of protons that give rise to Int_{IC}
- n_t – number of protons that give rise to Int_t
- Int_{IC} – integral of the internal calibrant resonance signal
- Int_t – integral of the target analyte resonance signal
- MW_{IC} – molecular weight of the internal calibrant
- MW_t – molecular weight of the target analyte
- m_{IC} – weight of the internal calibrant
- m_s – weight of the target analyte

Compounds ID	Purity (%)
**1**	99.71
**2**	99.36
**3**	99.75
**4**	99.96
**5**	99.80

Supplementary References

1. Stierand, K., Maass, P. C., & Rarey, M. Molecular complexes at a glance: automated generation of two-dimensional complex diagrams. *Bioinformatics* **22**, 1710-1716 (2006).
2. Wood, D. J., Korolchuk, S., Tatum, N. J., Wang, L. Z., Endicott, J. A., Noble, M. E. M., & Martin, M. P. Differences in the conformational energy landscape of CDK1 and CDK2 suggest a mechanism for achieving selective CDK inhibition. *Cell Chem. Biol.* **26**, 1-10 (2018).
