# Supplementary material for: Development of allosteric and selective CDK2 inhibitors for contraception with negative cooperativity to cyclin binding
Source: Nat Commun. 2023 Jun 3;14:3213. doi: 10.1038/s41467-023-38732-x (PMC10239507; doi:10.1038/s41467-023-38732-x)
Supplement: Supplementary file 2 — Reporting Summary [file 41467_2023_38732_MOESM2_ESM.pdf]

nature portfolio

Corresponding author(s): Gunda I. Georg

Last updated by author(s): 2023/05/04

Reporting Summary

Nature Portfolio wishes to improve the reproducibility of the work that we publish. This form provides structure for consistency and transparency in reporting. For further information on Nature Portfolio policies, see our Editorial Policies and the Editorial Policy Checklist.

Statistics

For all statistical analyses, confirm that the following items are present in the figure legend, table legend, main text, or Methods section.

n/a	Confirmed	
☐	☒	The exact sample size (n) for each experimental group/condition, given as a discrete number and unit of measurement
☐	☒	A statement on whether measurements were taken from distinct samples or whether the same sample was measured repeatedly
☐	☒	The statistical test(s) used AND whether they are one- or two-sided<br>*Only common tests should be described solely by name; describe more complex techniques in the Methods section.*
☒	☐	A description of all covariates tested
☒	☐	A description of any assumptions or corrections, such as tests of normality and adjustment for multiple comparisons
☐	☒	A full description of the statistical parameters including central tendency (e.g. means) or other basic estimates (e.g. regression coefficient) AND variation (e.g. standard deviation) or associated estimates of uncertainty (e.g. confidence intervals)
☐	☒	For null hypothesis testing, the test statistic (e.g. F, t, r) with confidence intervals, effect sizes, degrees of freedom and P value noted<br>*Give P values as exact values whenever suitable.*
☒	☐	For Bayesian analysis, information on the choice of priors and Markov chain Monte Carlo settings
☒	☐	For hierarchical and complex designs, identification of the appropriate level for tests and full reporting of outcomes
☒	☐	Estimates of effect sizes (e.g. Cohen's d, Pearson's r), indicating how they were calculated

Our web collection on statistics for biologists contains articles on many of the points above.

Software and code

Policy information about availability of computer code

Data collection	ImageJ 1.52a, ImageJ 1.53a
Data analysis	GraphPad Prism V9, GraphPad Prism V. 9.1.1. GraphPad Prism V. 9.3.0

For manuscripts utilizing custom algorithms or software that are central to the research but not yet described in published literature, software must be made available to editors and reviewers. We strongly encourage code deposition in a community repository (e.g. GitHub). See the Nature Portfolio guidelines for submitting code & software for further information.

Data

Policy information about availability of data

All manuscripts must include a data availability statement. This statement should provide the following information, where applicable:

- Accession codes, unique identifiers, or web links for publicly available datasets
- A description of any restrictions on data availability
- For clinical datasets or third party data, please ensure that the statement adheres to our policy

Source data are provided with this paper, including unprocessed blots, enzyme inhibition data, and CETSA data. The crystallographic data generated in this study are provided in the Supplementary Information. Accession codes for 7RWF and 7S84 have already been released. 8FOW, 8FP0 and 8FP5 will be released once the manuscript has been accepted/published.
7RWF [https://doi.org/10.2210/pdb7RWF/pdb] (Fo-wing)

7S84 [https://doi.org/10.2210/pdb7S84/pdb] (Fo-wing)
8FOW [https://doi.org/10.2210/pdb8FOW/pdb] (Fo-wing)
8FP0 [https://doi.org/10.2210/pdb8FP0/pdb] (Fo-wing)
8FP5 [https://doi.org/10.2210/pdb8FP5/pdb] (Fo-wing)

Human research participants

Policy information about studies involving human research participants and Sex and Gender in Research.

Reporting on sex and gender	NA
Population characteristics	NA
Recruitment	NA
Ethics oversight	*Identify the organization(s) that approved the study protocol.*

Note that full information on the approval of the study protocol must also be provided in the manuscript.

Field-specific reporting

Please select the one below that is the best fit for your research. If you are not sure, read the appropriate sections before making your selection.

☒ Life sciences ☐ Behavioural & social sciences ☐ Ecological, evolutionary & environmental sciences

For a reference copy of the document with all sections, see nature.com/documents/nr-reporting-summary-flat.pdf

Life sciences study design

All studies must disclose on these points even when the disclosure is negative.

Sample size	We used a sample size for each experiment that is common/standard for the method employed, whether it be n>3 for cellular and biochemical work or n=1-2 for biophysical measurements.
Data exclusions	No data were excluded from the analyses for the CETSA experiment.
Replication	The CETSA experiment was successfully replicated thrice independently. All attempts at independent replication for other experiments were successful, including twice for SPR experiments, thrice for ADP Glo experiments, once for ITC experiments, and once for FRET experiments
Randomization	Cells grown up for the CETSA experiments were randomly split for each condition. Randomization was conducted in the other experiments as applicable, whereas most experiments in the manuscript did not require randomization
Blinding	The CETSA experiments were conducted by one co-author, so blinding this person from the experimental conditions was not possible or generally necessary for these types of experiments. For other experiments in this work, blinding was not conducted for similar reasons as for the CETSA

Reporting for specific materials, systems and methods

We require information from authors about some types of materials, experimental systems and methods used in many studies. Here, indicate whether each material, system or method listed is relevant to your study. If you are not sure if a list item applies to your research, read the appropriate section before selecting a response.

Materials & experimental systems

n/a	Involved in the study	
☐	☒	Antibodies
☐	☒	Eukaryotic cell lines
☒	☐	Palaeontology and archaeology
☐	☒	Animals and other organisms
☒	☐	Clinical data
☒	☐	Dual use research of concern

Methods

n/a	Involved in the study	
☒	☐	ChIP-seq
☒	☐	Flow cytometry
☒	☐	MRI-based neuroimaging

Antibodies

Antibodies used	CETSA: anti-CDK2 (sc-6248, Santa Cruz Biotechnology, 1:2,000), anti-CDK1 (sc-54, Santa Cruz Biotechnology, 1:2,000) and anti-β-actin (A1978, Millipore Sigma, 1:2,000), secondary goat-anti-mouse (A16072, Invitrogen, 1:1,000 for CDK2 and CDK1 blots) and goat-anti-mouse (A32729, Invitrogen, 1:1,000 for β-actin blots) Alexa680 antibodies.<br><br>Cellular Cyclin E1 and CDK2 quantification: anti-cyclin E1 (#4129, Cell Signaling Technology, 1:2000), anti-CDK2 (sc-6248, Santa Cruz Biotechnology, 1:2000), and anti-β-actin (A1978, Millipore Sigma, 1:2,000), secondary horseradish peroxidase-conjugated-goat-anti-mouse (A16072, Invitrogen, 1:1,000 for cyclin E1 and CDK2 blots), Alexa680 conjugated-goat-anti-mouse (A32729, Invitrogen, 1:1,000 for β-actin blots) antibodies.<br><br>Immunofluorescent staining and analysis, CDK2 Western blot: anti-CDK2 (sc-6248, Santa Cruz Biotechnology,1:100), anti-SCP-3 monoclonal antibody (sc-74569) and anti-Cdk2 monoclonal antibody (sc-6248) Santa Cruz Biotechnology (Santa Cruz, CA). Anti-RAD51 polyclonal antibody (pc130) Millipore (Temecula, CA), anti-SCP1 polyclonal antibody (NB300-229) Novus (Centennial, CO), Alexa Fluor conjugated secondary antibodies (488 and 564; ab150073) Life Technologies (Grand Island, NY), The SPY1 antibody is non-commercial and was obtained from Angel Nebreda, which we acknowledged in the acknowledgements section.
Validation	All antibody reagents used were validated by the manufacturers.

Eukaryotic cell lines

Policy information about cell lines and Sex and Gender in Research

Cell line source(s)	Jurkat (ATCC) and OVCAR-3 (ATCC) for the CETSA experiment.
Authentication	The cell lines were purchased directly from ATCC and no authentification was performed.
Mycoplasma contamination	The cell lines were purchased directly from ATCC and were not tested for mycoplasma contamination.
Commonly misidentified lines<br>(See ICLAC register)	Not available for the CETSA experiment.

Animals and other research organisms

Policy information about studies involving animals; ARRIVE guidelines recommended for reporting animal research, and Sex and Gender in Research

Laboratory animals	Mouse, C57B6. 4 months old adult mice
Wild animals	No wild animals were used in this study
Reporting on sex	Experiments were done using testes tissue collected after euthanizing male mice. Only male meiosis was studied in the current manuscript and hence only male mice were used.
Field-collected samples	No field collected samples were used int his study
Ethics oversight	All rodent studies were approved by the Institutional Animal Care and Use Committee (IACUC) at KUM Medical Center.

Note that full information on the approval of the study protocol must also be provided in the manuscript.
